# Altered Expression of Ion Channels in White Matter Lesions of Progressive Multiple Sclerosis: What Do We Know About Their Function?

**DOI:** 10.3389/fncel.2021.685703

**Published:** 2021-06-25

**Authors:** Francesca Boscia, Maria Louise Elkjaer, Zsolt Illes, Maria Kukley

**Affiliations:** ^1^Division of Pharmacology, Department of Neuroscience, Reproductive and Dentistry Sciences, School of Medicine, University of Naples “Federico II”, Naples, Italy; ^2^Neurology Research Unit, Department of Clinical Research, University of Southern Denmark, Odense, Denmark; ^3^Department of Neurobiology Research, Institute of Molecular Medicine, University of Southern Denmark, Odense, Denmark; ^4^Department of Neurology, Odense University Hospital, Odense, Denmark; ^5^Achucarro Basque Center for Neuroscience, Leioa, Spain; ^6^Ikerbasque Basque Foundation for Science, Bilbao, Spain

**Keywords:** multiple sclerosis, progressive, white matter, lesions, ion channels, transcriptome

## Abstract

Despite significant advances in our understanding of the pathophysiology of multiple sclerosis (MS), knowledge about contribution of individual ion channels to axonal impairment and remyelination failure in progressive MS remains incomplete. Ion channel families play a fundamental role in maintaining white matter (WM) integrity and in regulating WM activities in axons, interstitial neurons, glia, and vascular cells. Recently, transcriptomic studies have considerably increased insight into the gene expression changes that occur in diverse WM lesions and the gene expression fingerprint of specific WM cells associated with secondary progressive MS. Here, we review the ion channel genes encoding K^+^, Ca^2+^, Na^+^, and Cl^−^ channels; ryanodine receptors; TRP channels; and others that are significantly and uniquely dysregulated in active, chronic active, inactive, remyelinating WM lesions, and normal-appearing WM of secondary progressive MS brain, based on recently published bulk and single-nuclei RNA-sequencing datasets. We discuss the current state of knowledge about the corresponding ion channels and their implication in the MS brain or in experimental models of MS. This comprehensive review suggests that the intense upregulation of voltage-gated Na^+^ channel genes in WM lesions with ongoing tissue damage may reflect the imbalance of Na^+^ homeostasis that is observed in progressive MS brain, while the upregulation of a large number of voltage-gated K^+^ channel genes may be linked to a protective response to limit neuronal excitability. In addition, the altered chloride homeostasis, revealed by the significant downregulation of voltage-gated Cl^−^ channels in MS lesions, may contribute to an altered inhibitory neurotransmission and increased excitability.

## Introduction

Multiple sclerosis (MS) is an inflammatory demyelinating disease of the central nervous system (CNS) affecting more than 2 million people worldwide. MS lesions in CNS white matter (WM) are multiple focal areas of myelin loss accompanied by inflammation, gliosis, phagocytic activity, and axonal damage (Compston and Coles, 2008; Kuhlmann et al., 2017; Filippi et al., 2018; Rommer et al., 2019). Available MS therapies have little benefit for secondary-progressive MS (SPMS) patients, who develop progressive disability after a disease course characterized by inflammatory attacks. Therefore, promoting neuroprotection and remyelination are important therapeutic goals to prevent irreversible neurological deficits and permanent disability.

Ion channels play a fundamental role in maintaining WM integrity and regulating function of axons, interstitial neurons (Sedmak and Judas, 2021), glia, and vascular cells. Dysregulation of ionic homeostasis in the WM during demyelination is decisive for axonal damage and cell death and may interfere with tissue repair processes (Boscia et al., 2020). Furthermore, MS may involve an acquired channelopathy (Waxman, 2001; Schattling et al., 2014). Hence, selectively targeting ion channels in WM represents an attractive strategy to overcome axonal and glial impairment and prevent disease progression.

**Figure 1:**
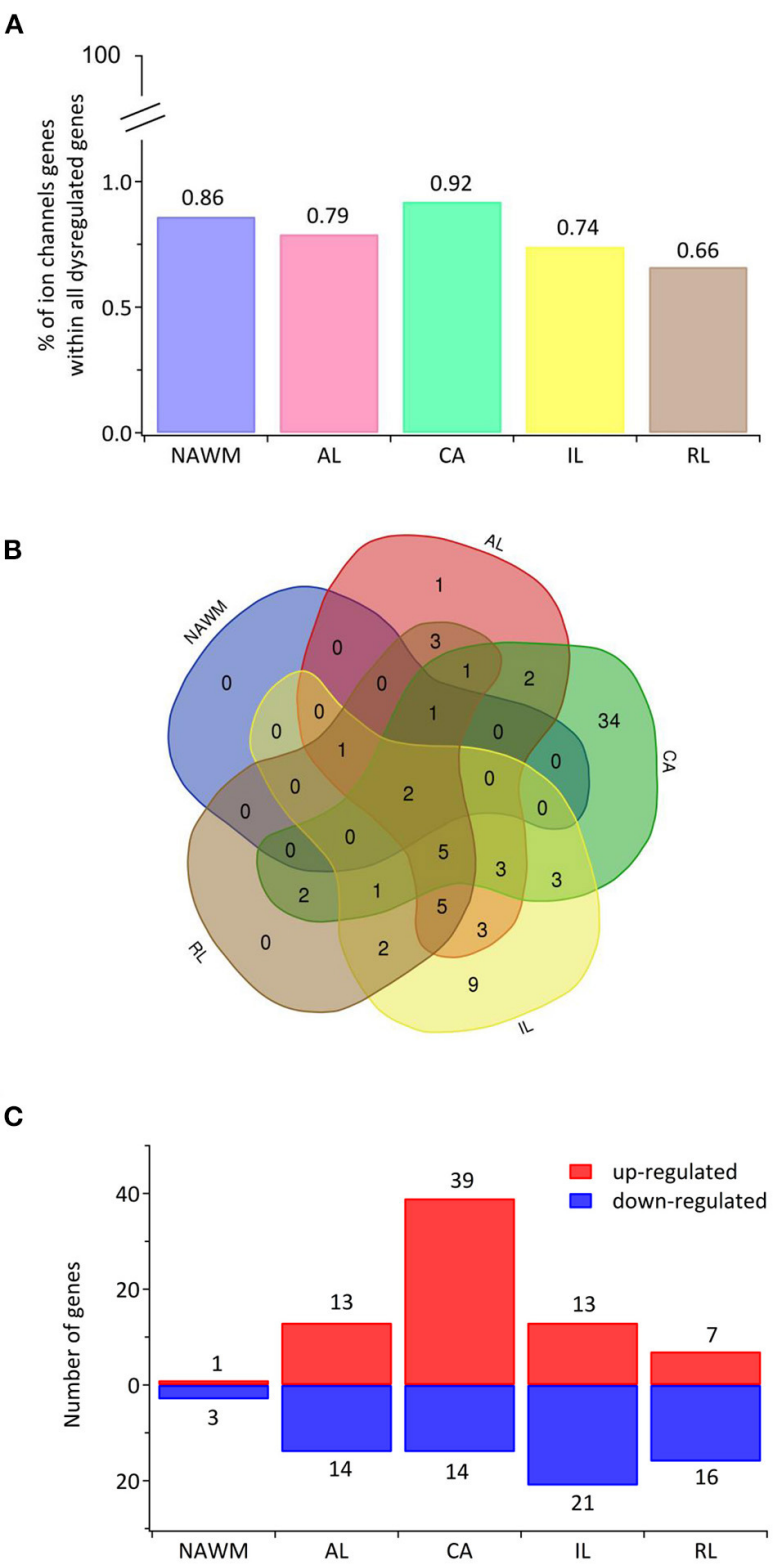
The transcriptional landscape of ion channels in different types of white matter brain lesions from patients with secondary progressive multiple sclerosis. **(A)** The percentage of significantly differentially expressed genes coding for ion channels among all dysregulated genes and within each lesion type [chronic active (CA), active (AL), inactive (IL), and remyelinating (RL)] and normal-appearing white matter (NAWM) compared to control white matter are indicated. **(B)** The Venn diagram shows the number of lesion-specific differentially expressed genes coding for ion channels and the number of overlapping genes among the lesion types. **(C)** The number of significantly differentially upregulated (red) and downregulated (blue) genes in each type of while matter lesion and NAWM compared to control white matter are indicated.

**Figure 2:**
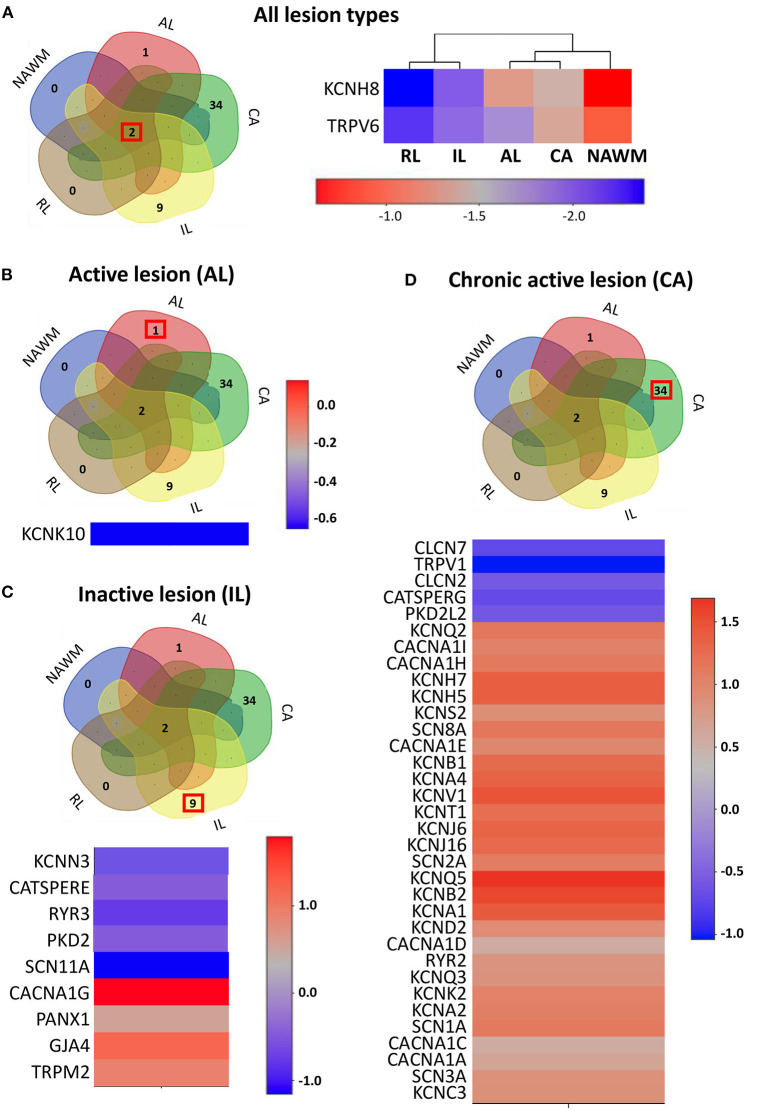
The expression profile of the ion channel genes uniquely expressed in different lesion types. **(A)** Left panel: The Venn diagram represents the number of overlapping and lesion-specific differentially expressed genes coding for ion channels in chronic active (CA), active (AL), inactive (IL), and remyelinating (RL) lesions and in normal-appearing white matter (NAWM) compared to control white matter. Right panel: The heatmap shows two genes, coding for ion channels KCNH8 and TRPV6 that are significantly altered in all lesion types compared to control white matter. Scale bar indicates fold changes. **(B)** The Venn diagram, the heatmap, and the scale bar show the single ion channel gene, KCNK10, which is uniquely downregulated in active lesion (AL). **(C)** The Venn diagram, the heatmap, and the scale bar show the eight genes coding for ion channels that are uniquely significantly differentially dysregulated in inactive lesion (IL). **(D)** The Venn diagram, the heatmap, and the scale bar show the 33 genes coding for ion channels that are significantly and differentially dysregulated compared to control white matter in chronic active lesion (CA). The red box in Venn diagrams marks the genes that are specifically dysregulated in the corresponding type of lesion.

**Figure 3:**
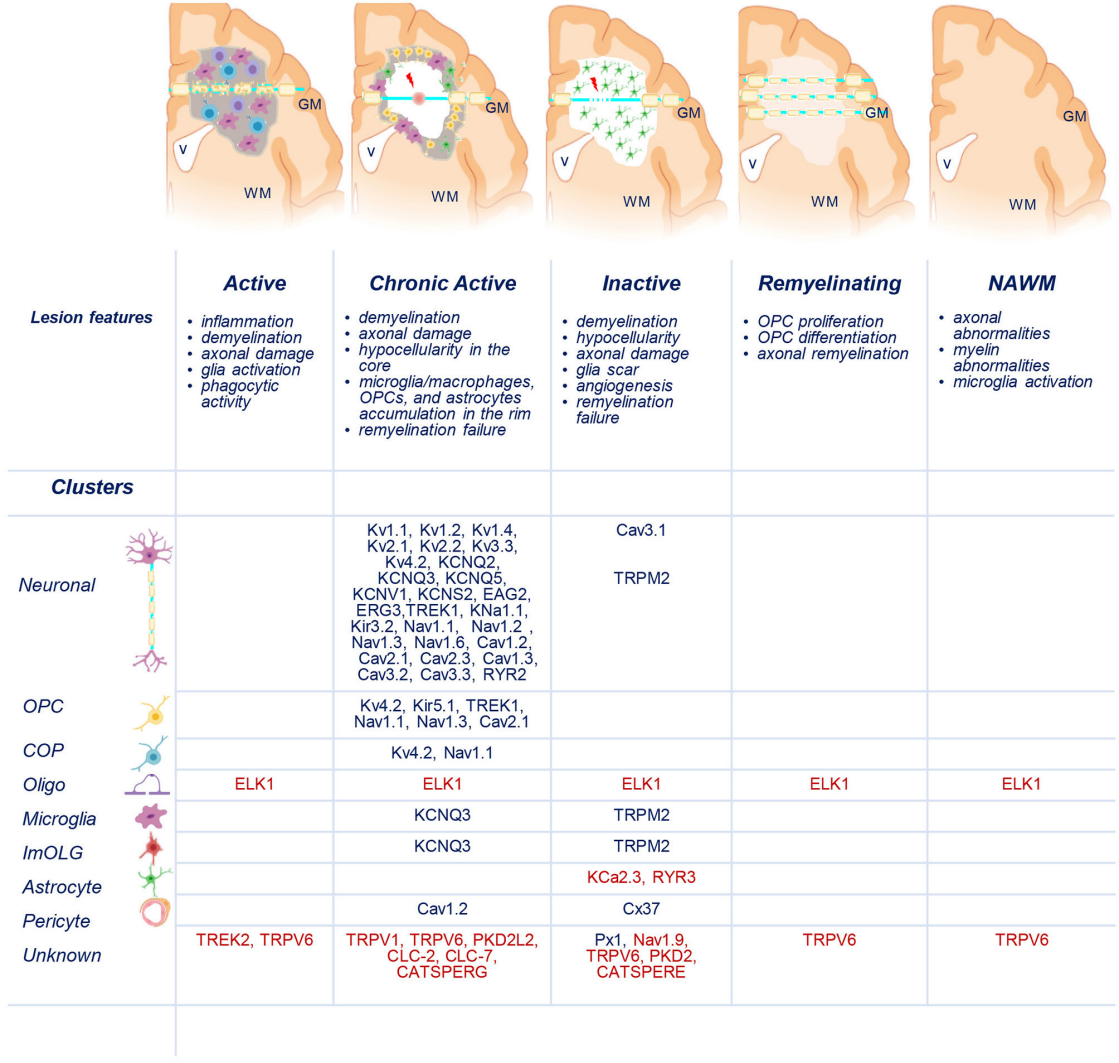
Distribution of uniquely dysregulated genes encoding ion channels in SPMS lesions. Schematic representation of active (AL), chronic active (CA), inactive (IL), and remyelinating (RL) lesions, and normal-appearing white matter (NAWM). Upregulated (blue) and downregulated (red) ion channels encoded by uniquely dysregulated genes are listed according to their expression in the lesions and in neuronal, oligodendrocyte precursor cells (OPCs), committed OPCs (COPs), oligodendrocytes (Oligo), microglia, immune oligo (ImOLG), astrocyte, pericyte, and unknown clusters. GM, gray matter; WM, white matter; v, brain ventricle. Gray areas indicate active inflammatory lesion, white areas indicate demyelinated inactive lesions, red spot indicates tissue damage, red arrow indicates axonal dysfunction. Source icon is from Biorender.com.

Recently, transcriptomic studies have considerably increased our insight into gene expression changes occurring in the MS brain (Elkjaer et al., 2019; Jakel et al., 2019; Schirmer et al., 2019). Aiming at identifying the ion channel genes governing WM dysfunction in SPMS brain, we analyzed the recent bulk RNA-sequencing (RNA-seq) datasets by using the MS-Atlas (Elkjaer et al., 2019; Frisch et al., 2020). We put a special emphasis on the distribution of shared and unique genes encoding ion channels in chronic active (CA), active (AL), inactive (IL), and remyelinating (RL) lesions, and normal-appearing white matter (NAWM) compared to control WM (Figures 1A,B, Table 1). We identified uniquely expressed ion channel genes: 34 genes in CA, 9 in IL, 1 in AL, as well as 2 genes in all lesions and NAWM (Figures 1, 2, Table 1). The CA lesions displayed the highest number of upregulated ion channels genes while downregulated ion channels genes were more consistently found in ILs (Figure 1C). Next, we explored recent single-nuclei RNA-seq (snRNA-seq) datasets to identify the expression of dysregulated ion channel genes in cell clusters in the WM of control and SPMS brain (Jakel et al., 2019; Tables 1, 2, Figure 3).

**Table 1:** Expression and distribution of unique and overlapping genes coding for ion channels within SPMS lesions.

**Protein**	**Gene**	**Bulk lesion^a^**	**Fold change Up (+)/down (–) regulated (compared to control WM)^a^**	**Current type/conductance**	**Highly expressed in WM clusters of human brain^b^**
**K**^**+**^ **channels**					
K_v_ 1.1	KCNA1	CA	**+1.42**	Delayed rectifier	
K_v_ 1.2	KCNA2	CA	**+1.06**	Delayed rectifier	neuron2
K_v_ 1.3	KCNA3	AL, CA, IL	**+1.67 (AL);** **+1.35 (CA);** **+1.34 (IL)**	Delayed rectifier	
K_v_ 1.4	KCNA4	CA	**+1.34**	A-type	
K_v_ 1.5	KCNA5	AL, RL	**+0.86 (AL);** **+1.36 (RL)**	Delayed rectifier	
K_v_ 2.2	KCNB2	CA	**+1.56**	Delayed rectifier	Neuron1, 2, 3, 4, 5
K_v_ 2.1	KCNB1	CA	**+1.26**	Delayed rectifier	Neuron1, 2, 3
K_v_ 3.3	KCNC3	CA	**+0.87**	A-type	
K_v_ 3.4	KCNC4	AL, IL	**+0.81 (AL);** **+0.72 (IL)**	A-type	
K_v_ 4.2	KCND2	CA	**+0.95**	A-type	OPC, COP, neuron1,3
K_v_ 4.3	KCND3	AL, CA, IL	**+0.63 (AL);** **+0.86 (CA);** **+0.93 (IL)**	A-type	neuron1, 2, 3
K_v_ 6.1	KCNG1	AL, RL	**+2.72 (AL);** **+3.7 (RL)**	Modifier of Kv 2	
K_v_ 7.1	KCNQ1	AL, CA	**+0.91 (AL);** **+0.75 (CA)**	M-type	
K_v_ 7.2	KCNQ2	CA	**+0.75**	M-type	neuron1, 2
K_v_ 7.3	KCNQ3	CA	**+0.85**	M-type	ImOLGs, neuron1, 2, 3, 5, microglia/macrophages
K_v_ 7.4	KCNQ4	AL, CA, IL, RL	**+1.19 (AL);+** **0.92 (CA);** **+1.36 (IL);** **+2.22 (RL)**	M-type	
K_v_ 7.5	KCNQ5	CA	**+1.69**	M-type	Neuron1, 2, 3, 5
K_v_ 8.1	KCNV1	CA	**+1.48**	Modifier of Kv 2	
K_v_ 9.2	KCNS2	CA	**+0.90**	Modifier of Kv 2	
K_v_ 9.3	KCNS3	AL, IL, RL, NAWM	−2.72 (AL); −1.5 (IL); −1.98 (RL); −0.71 (NAWM)	Modifier of Kv 2	
K_v_ 10.1/EAG1	KCNH1	CA, IL	**+0.81 (CA);** **+0.93 (IL)**	Delayed rectifier	Neuron1, 2, 3
K_v_ 10. 2/EAG2	KCNH5	CA	**+1.38**	Delayed rectifier	Neuron2
K_v_ 11.3/ERG3	KCNH7	CA	**+1.38**	Delayed rectifier	Neuron1, 2, 3, 5
K_v_ 12.1/ELK1	KCNH8	AL, CA, IL, RL, NAWM	−1.25 (AL); −1.4(CA); −2.05 (IL); −2.38 (RL); −0.62 (NAWM)	Delayed rectifier	Oligo3, Oligo4, Oligo6
TREK1	KCNK2	CA	**+1.03**	Leak, two pore	
TWIK2	KCNK6	AL, IL	**+1.57 (AL);** **+0.82 (IL)**	Leak, two pore	
TREK2	KCNK10	AL	−0.65	Leak, two pore	
K_Ca_1.1	KCNMA1	AL, CA, IL	**+0.69 (AL);** **+0.87 (CA);** **+0.7 (IL)**	Calcium-Activated	OPC, neuron1, 2, 3, 5, microglia/macrophages
K_Ca_2.3	KCNN3	IL	−0.7	Calcium-Activated	Astrocytes1
K_Na_1.1	KCNT1	CA	**+1.24**	Sodium-Activated	
K_Na_1.2	KCNT2	CA, IL	**+0.92 (CA);** **+1.15 (IL)**	Sodium-Activated	Neuron1, 2, 3, pericytes, vascular smooth cells
K_ir_2.1	KCNJ2	AL, CA, IL, RL	−0.54 (AL); −0.48 (CA); −0.54 (IL); −0.92 (RL)	Inward rectifier	
K_ir_3.4	KCNJ5	AL, CA, RL, NAWM	**+2.58 (AL);** **+1.56 (CA);** **+1.9 (RL);** **+1.53 (NAWM)**	Inward rectifier	
K_ir_3.2	KCNJ6	CA	**+1.34**	Inward rectifier	Neuron1, 2, 3
K_ir_6.1	KCNJ8	AL, IL	**+0.74 (AL);** **+0.71 (IL)**	Inward rectifier	
K_ir_3.3	KCNJ9	CA, RL	−0.52 (CA); −0.9 (RL)	Inward rectifier	
K_ir_4.1	KCNJ10	IL, RL	−1.06 (IL); −1.09 (RL)	Inward rectifier	Oligo5
K_ir_5.1	KCNJ16	CA	**+1.27**	Inward rectifier	
**Na**^**+**^ **channels**					
Na_v_1.1	SCN1A	CA	**+1.12**	TTX-sensitive	OPC, COP, neuron1, 2, 3, 4, 5
Na_v_1.2	SCN2A	CA	**+1.1**	TTX-sensitive	Neuron1, 2, 3, 4, 5
Na_v_1.3	SCN3A	CA	**+0.87**	TTX-sensitive	OPC, neuron1, 2, 3, 5
Na_v_1.6	SCN8A	CA	**+1.15**	TTX-sensitive	Neuron1, 2, 3, 5
Na_v_1.9	SCN11A	IL	−1.16	TTX-resistant	
**Ca**^**2+**^ **channels**					
Ca_v_1.2	CACNA1C	CA	**+0.56**	L-type	Neuron1, 2, 3, 5, pericytes
Ca_v_1.3	CACNA1D	CA	**+0.57**	L-type	Neuron1,3
Ca_v_2.1	CACNA1A	CA	**+0.64**	P/Q-type	OPC, neuron1, 2
Ca_v_2.3	CACNA1E	CA	**+0.97**	P/Q-type	Neuron1, 2, 5
Ca_v_3.1	CACNA1G	IL	**+1.8**	T-type	
Ca_v_3.2	CACNA1H	CA	**+1.12**	T-type	
Ca_v_3.3	CACNA1I	CA	**+1.03**	T-type	
**Ryanodine**					
Ryr2	RYR2	CA	**+0.85**	Ca^2+^ Release channel	Neuron1, 2, 3
Ryr3	RYR3	IL	−0.76	Ca^2+^ Release channel	Astrocytes1
**TRP channels**					
TRPC1	TRPC1	AL, IL, RL	−0.5 (AL); −0.48 (IL); −0.85 (RL)	Ca^2+^-permeable cation channel	
TRPM2	TRPM2	IL	**+0.92**	Ca^2+^-permeable cation channel	
TRPM3	TRPM3	IL, RL	−1.09 (IL); −0.98 (RL)	Ca^2+^-permeable cation channel	Astrocytes1, neuron1
TRPM6	TRPM6	CA, IL, RL	−0.99 (CA); −1.06 (IL); −1.08 (RL)	Ca^2+^-permeable cation channel	
TRPP1	PKD2	IL	−0.48	Ca^2+^-permeable cation channel	
TRPP3	PKD2L2	CA	−0.58	Ca^2+^-permeable cation channel	
TRPV1	TRPV1	CA	−1.04	Ca^2+^-permeable cation channel	
TRPV3	TRPV3	AL, CA, IL, RL	−0.51 (AL); −0.72 (CA); −0.5 (IL); −0.74 (RL)	Ca^2+^-permeable cation channel	
TRPV5	TRPV5	AL, CA, IL, RL	−1.4 (AL); −1.67 (CA); −1.72 (IL); −2.02 (RL)	Ca^2+^-permeable cation channel	
TRPV6	TRPV6	AL, CA, IL, RL, NAWM	−1.77 (AL); −1.97 (IL); −1.32 (CA); −2.23 (RL); 0.86 (NAWM)	Ca^2+^-permeable cation channel	
**Cl**^**−**^ **channels**					
CLC-2	CLCN2	CA	−0.57	Inward rectification	
CLC-4	CLCN4	AL, IL, RL	−0.79 (AL); −0.73 (IL); −1.03 (RL)	Cl^−^/H^+^ antiporter	
CLC-7	CLCN7	CA	−0.72	Cl^−^/H^+^ antiporter	
**Connexins and pannexins**					
Cx43	GJA1	AL, CA, RL	**+1.53 (AL);** **+1.12 (CA);** **+1.19 (RL)**	Monovalent and divalent ions	Astrocytes1, astrocytes2
Cx32	GJB1	AL, CA, IL, RL	−1.6 (AL); −1.5 (CA); −1.85 (IL); −2.44 (RL)	Monovalent and divalent ions	Oligo5
CX37	GJA4	IL	**+1.19**	Monovalent and divalent ions	Pericytes
Cx47	GJC2	AL, CA	−1.62 (AL); −1.74 (CA)	Monovalent and divalent ions	
Panx1 **Others**	PX1	IL	**+0.56**	Monovalent and divalent ions	
Piezo2	PIEZO2	AL, CA, IL, RL	−0.92 (AL); −1.01 (CA); −0.93 (IL); −1.49 (RL)	Ca^2+^ -permeable	Oligo1, Oligo6
CFTR	CFTR	AL, CA, IL, RL	−1.22 (AL); −1.37 (CA); −1.77 (IL); −1.86 (RL)	Cl^−^-permeable	Oligo1
H_v_1	HVCN1	CA, RL	**+0.71 (CA);** **+0.92 (RL)**	H^+^-selective	
Na_vi_2.1	NALCN	AL, IL, RL	−0.49 (AL); −0.73 (IL); −0.88 (RL)	Sodium leak channel, non-selective	
Orai3	ORAI3	AL, RL	**+0.87 (AL);** **+1.25 (RL)**	Store-Operated Ca^2+^ entry	
Aquaporin 1	AQP1	CA, IL	−1.03 (CA); −0.12 (IL)	Water, ammonia, H_2_0_2_ permeability	Astrocytes1, astrocytes2
CATSPERG	CATSPERG	CA	**+0.7**	Ca^2+^ -permeable	
CATSPERE	CATSPERE	IL	−0.48	Ca^2+^ -permeable	

a *Expression and distribution of unique and overlapping genes coding for ion channels within chronic active (CA), active (AL), inactive (IL) remyelinating (RL) lesions, and normal-appearing white matter (NAWM). The information is based on the bulk-RNAseq (Elkjaer et al., 2019) and data are collected from the database available at www.msatlas.dk (Frisch et al., 2020)*.

b *Cell type-specific clusters with significant expression of ion channels genes in human brain WM. The information is based on the snRNAseq from the WM of individuals with SPMS and non-neurological control subjects (Jakel et al., 2019), and data are collected from the database available at https://ki.se/mbb/oligointernodeen/ where the encoded subunits were listed according to the IUPHAR nomenclature. Fold changes of up-regulated genes are shown in bold*.

**Table 2:** Profiling expression of unique gene in lesions in WM clusters of healthy and SPMS brain^a^.

**Protein**	**Gene**	**Neuron**	**Astrocyte**	**OPC**	**COP**	**ImOLG**	**Oligo**	**Microglia**	**Pericyte**
**K**^**+**^ **channels**									
Kv1.1	KCNA1	n.d	n.d	n.d	n.d	n.d	n.d	n.d	n.d
Kv1.2	KCNA2	+	+/–	+/–	+/–	–	+/–	–	–
Kv1.4	KCNA4	+/–	–	–	–	–	–	–	–
Kv2.1	KCNB1	+	+/–	+/–	+	+/–	+/–	–	+
Kv2.2	KCNB2	++	+/–	–	+/–	+/–	+/–	–	–
Kv3.3	KCNC3	+	+/–	–	+/–	+/–	+/–	+/–	–
Kv4.2	KCND2	+++	+	++++	+++	+	+/–	+/–	+/–
Kv7.2	KCNQ2	+	+/–	+	+	+/–	+/–	–	+/–
Kv7.3	KCNQ3	+++	+	+	+	++	+/–	+++	+/–
Kv7.5	KCNQ5	++++	+	+/–	+	+	+/–	+/–	+/–
Kv8.1	KCNV1	+	–	–	+/–	+/–	–	–	–
Kv9.2	KCNS2	+	–	–	+/–	–	–	–	–
Kv10. 2/EAG2	KCNH5	+	+/–	+/–	+	+/–	+/–	–	–
Kv11.3/ERG3	KCNH7	+++	+/–	–	+	+	+/–	–	–
Kv12.1/ELK1	KCNH8	+	+++	++	+++	++	+++	+/–	+/–
TREK1	KCNK2	+	+/–	+	+/–	–	–	–	–
TREK2	KCNK10	+	+/–	+/–	+	+/–	+/–	–	–
K_Ca_2.3	KCNN3	+	++	+	+	+	+/–	+/–	+/–
KNa1.1	KCNT1	+	–	–	+/–	+/–	–	–	–
Kir3.2	KCNJ6	+	+/–	+	+	–	+	–	–
Kir5.1	KCNJ16	–	+/–	+	+	–	–	–	–
**Na**^**+**^ **channels**									
Nav1.1	SCN1A	++	+	+++	++	+	+/–	–	–
Nav1.2	SCN2A	+++	+	+/–	+	+	+/–	–	+/–
Nav1.3	SCN3A	++	+/–	++	++	+	+	–	+/–
Nav1.6	SCN8A	n.d	n.d	n.d	n.d	n.d	n.d	n.d	n.d
Nav1.9	SCN11A	n.d	n.d	n.d	n.d	n.d	n.d	n.d	n.d
**Ca**^**2+**^ **channels**									
Cav1.2	CACNA1C	+++	+	+	+	+	+/–	–	+++
Cav1.3	CACNA1D	++	+/–	+	+	+	+/–	+	–
Cav2.1	CACNA1A	+++	+	+++	++	+	+/–	+	+/–
Cav2.3	CACNA1E	++	+/–	+/–	+	+	+/–	–	–
Cav3.1	CACNA1G	+	–	+/–	+/–	–	–	–	–
Cav3.2	CACNA1H	n.d	n.d	n.d	n.d	n.d	n.d	n.d	n.d
Cav3.3	CACNA1I	+	–	–	+/–	–	–	–	–
**Ryanodine**									
Ryr2	RYR2	++++	+	+/–	+	+	+	+/–	+
Ryr3	RYR3	+	+++	+	+	+	+/–	+/–	+/–
**TRP channels**									
TRPM2	TRPM2	+	+/–	–	+/–	+	–	+	+/–
TRPP1	PKD2	+	++	+	++	++	+	+	+
TRPP3	PKD2L2	n.d	n.d	n.d	n.d	n.d	n.d	n.d	n.d
TRPV1	TRPV1	+/–	+/–	–	+/–	–	+/–	–	–
TRPV6	TRPV6	n.d	n.d	n.d	n.d	n.d	n.d	n.d	n.d
**Cl**^**−**^ **channels**									
CLC−2	CLCN2	+/–	+/–	+/–	+/–	+/–	+	–	–
CLC−7	CLCN7	+	+	+	+	+	+	+	+/–
**Connexins**									
Cx37	GJA4	–	–	–	–	–	–	–	++
**Pannexin**									
Px1	PANX1	n.d	n.d	n.d	n.d	n.d	n.d	n.d	n.d
**Catsper**									
CATSPERG	CATSPERG	+/–	+/–	–	+/–	+/–	+/–	–	–
CATSPERE	CATSPERE	n.d	n.d	n.d	n.d	n.d	n.d	n.d	n.d

a *sn-RNAseq from the white matter of individuals with SPMS and non-neurological controls. The information is based on the snRNAseq from the WM of individuals with SPMS and non-neurological control subjects (Jakel et al., 2019), and data are collected from the database available at https://ki.se/mbb/oligointernodeen/. Expression levels are based on the mean normalized expression counts (log-scale) per cluster*.

*+/– (log scale > 0.01 ≤ 0.1); + (log scale > 0.1 ≤ 0.5); ++ (log scale > 0.6 ≤ 1); +++ (log scale >1.1 ≤ 1.5); ++++ (log scale > 1.5); – (log scale 0); n.d., not detected; red (+), highly expressed gene in the cluster if compared to the rest of the clusters*.

*Note, that in the database, some of the clusters encompass both control and MS samples and, therefore, the mean can represent a combination of counts from control and MS brain*.

**Figure 4:**
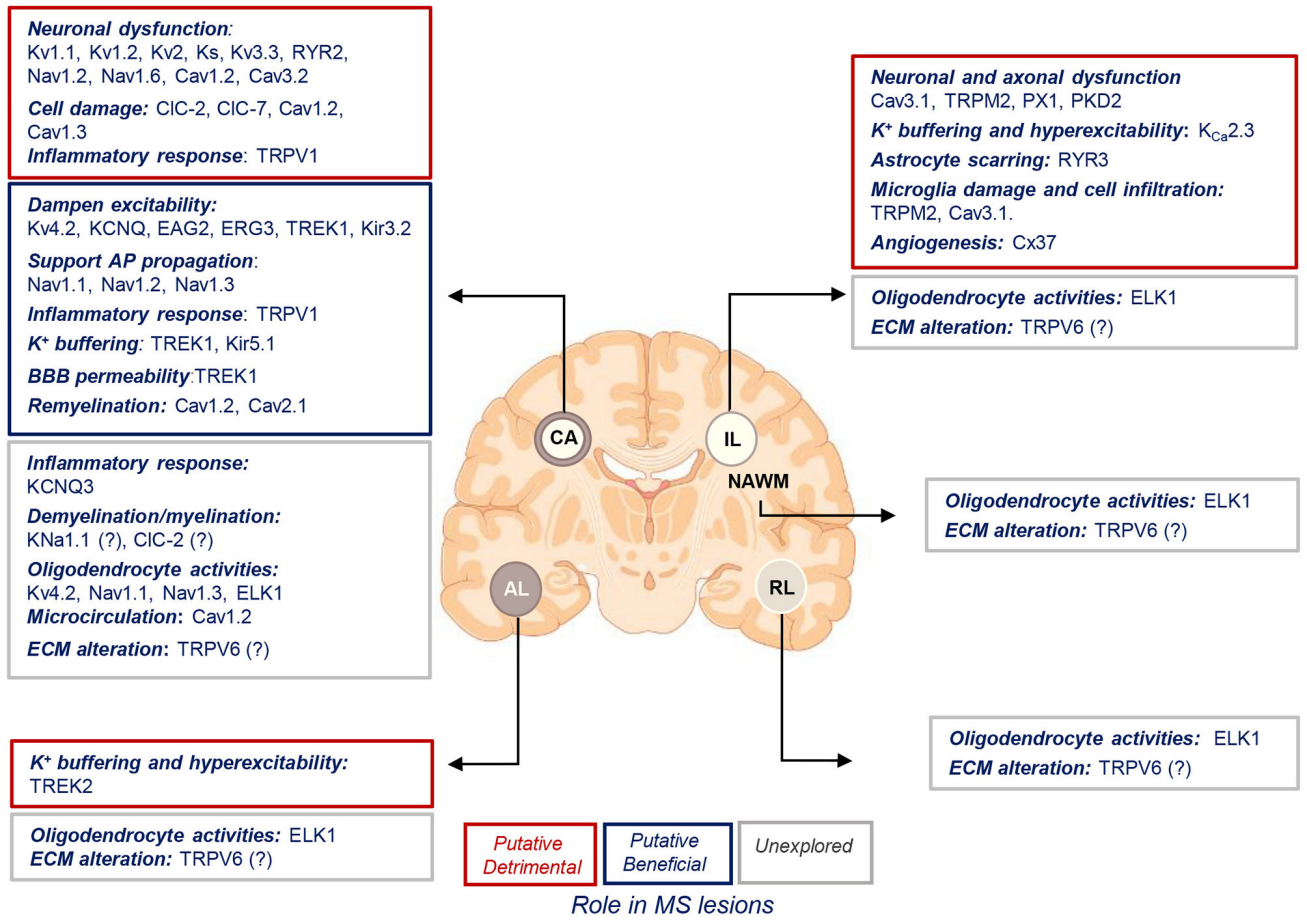
Putative roles of ion channels encoded by uniquely dysregulated genes in SPMS lesions. Schematic illustration of the putative detrimental (red box), putative beneficial (blue box), and unexplored (gray box) functional roles of ion channel encoded by the uniquely dysregulated genes in active (AL), chronic active (CA), inactive (IL), and remyelinating (RL) lesions, and normal-appearing white matter (NAWM) of SPMS brain. Source icon is from Biorender.com.

The goal of the present review is to discuss the current knowledge on the expression and function of ion channels that turned out to be significantly and uniquely dysregulated in WM lesions of SPMS brain. We summarize the information in the context of human MS and the related experimental models (Tables 1–3, Figure 4).

**Table 3:** Expression and role of unique dysregulated ion channels in experimental models of MS.

**Gene/protein**	**Distribution, localization**	**Cellular functions during physiological conditions**	**WM in MS models**	
			**Alterations**	**Role**
KCNA1/K_v_1.1	JPN of myelinated axons	Regulate AP propagation and neural excitability	Redistribution to internodes and nodal segments, upregulation	Hyperpolarise axonal V_rest_, affect AP threshold, impair AP conduction
	Microglia, astrocyte (t), OPCs (t)	Proliferation, cell activation		
KCNA2/K_v_1.2	JPN of myelinated axons	Regulate AP propagation and neural excitability	Redistribution to internodes and nodal segments, upregulation	Hyperpolarise axonal V_rest_, affect AP threshold, impair AP conduction
	Reactive astrocyte, microglia, OPC	Proliferation, cell activation		
KCNA4/K_v_1.4	Axons (HP)	Regulate AP propagation and neural excitability	Upregulation in astrocytes and OPCs around EAE lesions	Deficiency ameliorated EAE course in KO mice, but have no effect on demyelination/remyelination in the cuprizone model
	Reactive astrocyte, OPCs	Proliferation		
KCNB1/K_v_2.1	Soma, proximal dendrites, AIS Microglia, OPCs (t)	Influence AP duration during high frequency firing, regulate neuronal excitability	Unknown in WM Downregulation in motor neurons of GM spinal cord during EAE	Unknown
KCNB2/K_v_2.2	Soma, proximal dendrites, AIS Not detected in glia	Influence AP duration during high frequency firing, regulate neuronal excitability	Unknown	Unknown
KCNC3/K_v_3.3	Axons, somatodendritic compartment Astrocyte, microglia (t), OPCs (t)	Regulate AP firing at high frequency	Upregulation in some injured WM axons	Unknown
KCND2/K_v_4.2	Soma, dendrites Astrocyte (t), OPCs (t), microglia (t)	Regulate threshold for AP initiation and repolarization, frequency-dependent AP broadening, AP back-propagation	Unknown	Unknown
KCNQ2/K_v_7.2	AIS, nodes of Ranvier OPCs, microglia	Stabilize V_rest_, regulate activity of Na_V_-channels, accelerate AP upstroke, influence neuronal subthreshold excitability, regulate spike generation, and repetitive firing	Unknown	Unknown
KCNQ3/K_v_7.3	AIS, nodes of Ranvier Microglia (pro-inflammatory), OPCs, astrocyte (t)	Stabilize V_rest_, regulate activity of Na_V_-channels, accelerate AP upstroke, influence neuronal subthreshold excitability, regulate spike generation and repetitive firing	Unknown in WM Upregulated in demyelinated neocortical axons of L5 pyramidal neurons in the cuprizone model.	Unknown in WM Ensure AP conduction in demyelinated GM axons, decrease excitability
KCNQ5/K_v_7.5	Soma, dendrites Astrocyte, OPCs, microglia	Contributes to AHP currents in the HP	Unknown	Unknown
KCNV1/K_v_8.1	Unknown Oligo lineage (t)	Co-assemble with K_v_2.1, reduce K_v_2.1 current density which may lead to AP broadening and hyper-synchronized high-frequency firing	Unknown	Unknown
KCNS2/K_v_9.2	Unknown Oligo lineage (t)	Co-assemble with K_v_2.1	Unknown	Unknown
KCNH5/EAG2	Unknown Astrocyte (t), OPCs (t)	Unknown	Unknown	Unknown
KCNH7/ERG3	Unknown Astrocyte (t), OPCs (t), microglia (t)	Dampen excitability, stabilize V_rest_	Unknown	Unknown
KCNH8/ELK1	Unknown OPCs (t)	Unknown	Unknown	Unknown
KCNK2/TREK1	Axons, and node of Ranviers in afferent myelinated nerve Astrocyte, microglia (t) OPCs (t)	Contribute to “leak” K^+^-current, help establishing and maintaining V_rest_, regulate neuronal excitability, ensure AP repolarization at nodes of Ranvier in afferent myelinated fibers Contribute to passive membrane K^+^ conductance, glutamate release	Unknown	Deficiency aggravates EAE course in KO mice Channel activation reduces CNS immune cell trafficking across BBB and attenuate EAE course
KCNK10/TREK2	Unknown Astrocyte OPCs (t)	Contribute to “leak” K^+^-current, help establishing and maintaining V_rest_ Contribute to K^+^ buffering, glutamate clearance	Unknown	Unknown
KCNT1/K_Na_1.1	Soma, axons Astrocytes (t)	Regulate the generation of slow afterhyperpolarization, firing patterns, and setting and stabilizing the V_rest_	Unknown	Unknown
KCNN3/K_Ca_2.3	Dendrites, AIS Astrocyte, microglia, oligo lineage (t)	Regulate AP propagation and neuronal excitability, contribute to maintaining Ca^2+^-homeostasis K^+^ buffering in astrocytes Microglia proliferation and cytokines production	Unknown	Unknown
KCNJ6/K_ir_3.2	Somatodendritic compartment Astrocyte, oligo lineage (t)	K^+^-homeostasis, maintenance of V_rest_, hyperpolarization, control of AP firing and neuronal excitability, inhibition of excitatory neurotransmitter release	Unknown	Unknown
KCNJ16/K_ir_5.1	Somatodendritic compartment, dendritic spines Astrocyte, oligo lineage, microglia (t)	Silent channel when combined with K_ir_2.1. When combined with K_ir_4.1, build channels with larger conductance and greater pH-sensitivity. Plays a role in synaptic transmission Chemoreception K^+^ buffering	Unknown	Unknown
SCN1A/Na_v_1.1	Somatodendritic compartment, AIS, nodes of Ranvier Microglia, astrocyte, OPCs (t)	Saltatory conduction, maintenance of sustained firing, control of excitability Microglia phagocytosis, cytokine release	Increase or no change; localize along the demyelinated regions	Unknown
SCN2A/Na_v_1.2	AIS, immature nodes of Ranvier, along the non-myelinated axons Astrocyte, pre-oligodendrocytes	Back-propagation of AP into the somatodendritic compartment, may support slow spike propagation Oligo maturation	Increase of diffuse distribution along demyelinated axons in various mouse models; no change in myelin-deficient rat Upregulated in astrocytes during EAE	Unclear. Suggested: preservation of AP propagation, or axonal damage
SCN3A/Na_v_1.3	Somatodendritic compartment, along the axons including myelinated fibers Astrocyte oligo lineage (t)	AP initiation and propagation, proliferation and migration of cortical progenitors	No change in the optic nerve	Unknown
SCN8A/Na_v_1.6	AIS, nodes of Ranvier; low density on cell soma, dendritic shafts, synapses Astrocyte, microglia oligo (t)	AP initiation and propagation, neuronal excitability	Decrease at the nodes of Ranvier, increase of diffuse distribution along the damaged axons, no change at AIS Upregulated in microglia/macrophages during EAE	May trigger Na^+^ increase in axoplasm, reversal of NCX, and intra-axonal Ca^2+^ overload. Deletion improves axonal health during EAE
SCN11A/Na_v_1.9	Soma, proximal processes Negligible in all glial cells (t)	Regulate excitation, control activity-dependent axonal elongation, mediate sustained depolarizing current upon activation of muscarinic receptors	Unknown	Unknown
CACNA1C/Ca_V_1.2	Somatodendritic compartment (synaptically, extrasynaptically), axons, axonal terminals (extrasynaptically), pioneer axons during development Astrocyte, oligo lineage, reactive microglia	Synaptic modulation, propagation of dendritic Ca^2+^ spikes, regulation of glutamate receptor trafficking, CREB phosphorylation, coupling of excitation to nuclear gene transcription, modulation of long-term potentiation, neurites growth and axonal pathfinding during development Astrogliosis OPCs development and myelination	Unknown	Unknown. Suggested: Neurodegeneration because L-type VGCCs blockers attenuate mitochondrial pathology in nerve fibers and axonal loss Deletion in astrocyte- reduces cell activation and pro-inflammatory mediators release in the cuprizone model Deletion in OPCs reduced remyelination in the cuprizone model
CACNA1D/Ca_V_1.3	Somatodendritic compartment, axonal cylinders Astrocyte, microglia oligo lineage	Pacemaking activity, spontaneous firing, Ca^2+^-dependent post-burst after-hyperpolarization, Ca^2+^-dependent intracellular signaling pathways, regulation of morphology of dendritic spines and axonal arbores Oligodendrocyte-axon signaling, release of pro-inflammatory mediators by microglia	Unknown	Unknown. Suggested: neuroprotection because L-type VGCCs blockers attenuate mitochondrial pathology in nerve fibers and axonal loss
CACNA1A/Ca_V_2.1	Axonal synaptic terminals, axonal shafts in WM, somatodendritic compartment Reactive astrocyte OPCs, premyelinating oligo, microglia (t)	Neurotransmitter release at neuronal and neuron-glia synapses, regulation of BK and SK channels, control of neuronal firing, regulation of gene expression, local Ca^2+^ signaling, and cell survival Calcium influx in oligo upon neuronal activity	Unknown	Unknown
CACNA1E/Ca_V_2.3	Dendritic spines, axonal terminals Astrocyte, oligodendrocyte	Neurotransmitter release, synaptic plasticity, regulation of BK, SK, and K_V_4.2 channels	Unknown	Unknown
CACNA1G/Ca_V_3.1	Somatodendritic compartment, AIS Astrocyte (t) oligo lineage	Generation and timing of APs, regulation of neuronal excitability, rhythmic AP bursts in thalamus, neuronal oscillations, neurotransmitter release	Unknown	T-cells from KO mice show decreased cytokine release Deficiency in KO mice inhibits the autoimmune response in the EAE model
CACNA1H/Ca_V_3.2	Somatodendritic compartment, AIS Astrocyte oligo lineage	Generation and timing of APs, regulation of neuronal excitability, rhythmic AP bursts in thalamus, neuronal oscillations, neurotransmitter release	Unknown	Unknown
CACNA1I/Ca_V_3.3	Somatodendritic compartment	Generation and timing of APs, regulation of neuronal excitability, rhythmic AP bursts in thalamus, neuronal oscillations, neurotransmitter release	Unknown	Unknown
RyR2	Along ER (also in axons) Astrocyte, oligo lineage	Ca^2+^ release from the ER into the cytoplasm, vesicle fusion, neurotransmitter release, synaptic plasticity, growth cone dynamics	Unknown	Unknown
RyR3	Along ER (also in axons) Astrocyte, OPCs, oligodendrocytes	Ca^2+^ release from the ER into the cytoplasm, vesicle fusion, neurotransmitter release, synaptic plasticity, growth cone dynamics Astrocyte motility OPCs development	Unknown	Unknown
TRPV1	Soma, post-synaptic dendritic spines, synaptic vesicles Astrocyte, microglia, oligodendrocytes	Regulation of Ca^2+^-signaling, synaptic plasticity Astrocyte: migration, chemotaxis, activation during stress, inflammasome activation Microglia: migration, cytokine production, ROS generation, phagocytosis, polarization, cell death	Suggested a main role in regulating microglia inflammatory response	Both detrimental and beneficial effects have been described in EAE disease
TRPV6	Unknown Astrocyte (t)	Unknown	Unknown	Unknown
TRPM2	Soma and neurites in neuronal cultures Microglia, astrocyte (t), oligodendrocyte (t)	Contribute to synaptic plasticity and play an inhibitory role in neurite outgrowth Microglia activation and generation of proinflammatory mediators	Upregulated in monocyte-lineage cells	TRPM2 deficiency reduce monocyte infiltration in EAE
PKD2/TRPP1	ER, primary cilia, and plasma membrane Astrocyte (t), microglia (t), oligo lineage (t)	Maintenance of Ca^2+^-homeostasis, cell proliferation	Unknown	Unknown
PKD2L2/TRPP3	Unknown Astrocyte (t), microglia (t)	Unknown	Unknown	Unknown
CLCN2/CLC-2	Plasma membranes, intracellular membranes Astrocyte, OPCs, microglia	Maintenance of low intracellular Cl^−^ level, control of cell volume homeostasis, regulation of GABA_A_R-mediated synaptic inputs, regulation of neuronal excitability Interacts with AQP4 in astrocytes, regulates OPCs differentiation, contribute to volume regulation and phagocytosis in microglia	Unknown	Unknown
CLCN7/CLC-7	Lysosomes Microglia, astrocyte (t), oligo lineage (t)	Suggested function in the neuronal endo-lysosomal pathway Regulate lysosomal acidification in activated microglia	Unknown	Unknown
GJA4/CX37	Largely expressed in vascular cells	Regulate vasomotor activity, endothelial permeability, and maintenance of body fluid balance	Unknown	Unknown
PANX1/Px1	Soma, dendrites, axons Astrocyte, OPCs microglia	Paracrine and autocrine signaling, ATP-sensitive ATP release in complex with P2X_7_Rs, intercellular propagation of Ca^2+^-waves, cell differentiation, migration, synaptic plasticity, memory	Unknown	Panx-1 induced ATP release and inflammasome activation contribute to WM damage during EAE Inhibition of Panx1 using pharmacology or gene disruption delays and attenuates disease course in EAE and cuprizone model
CATSPERG	Unknown Oligo lineage (t) Microglia (t)	Unknown	Unknown	Unknown
CATSPERE	Unknown	Unknown	Unknown	Unknown

*AHP, afterhyperpolarization; AIS, axon initial segment; AP, action potential; BK, big-conductance Ca^2+^-activated K^+^-channels; ER, endoplasmatic reticulum; GABA_A_R, ionotropic gamma aminobutyric acid A receptor; HP, hippocampus; JPN, juxtaparanodal regions; NCX, Na^+^/Ca^2+^ exchanger; SCI, spinal cord injury; SK, small-conductance Ca^2+^-activated K^+^-channels; SSCx, somatosensory cortex; t, transcipts; V_rest_, resting membrane potential*.

## K^+^ Channels

### Voltage-Gated K^+^ Channels (K_v_)

K_v_ channels are composed of four α-subunits that assemble as homo- or hetero-tetramers to form a membrane pore. Forty human genes encode for K_v_ α-subunits representing 12 families. K_V_1–K_v_4 (Shaker, Shab, Shaw, and Shal), K_V_7 (KCNQ), and K_V_10–K_V_12 (eag, erg, and elk) α-subunits produce functional channels, while K_v_5, K_v_6, K_v_8, and K_v_9 fail to produce currents when expressed alone in heterologous expression system and are considered modulatory subunits for K_v_2-subfamily. The diversity of K_v_ channels is further increased by the ability of α-subunits to combine with auxiliary subunits, which regulate gating properties.

#### K_v_1.1, K_v_1.2, and K_v_1.4 (KCNA1, KCNA2, and KCNA4)

*KCNA* genes encode for low-threshold voltage-activated K_v_1 (K_v_1.1–1.8) channels, of which K_v_1.1–K_v_1.6 are expressed in the brain (Chittajallu et al., 2002; Vautier et al., 2004; Vacher et al., 2008; Rasmussen and Trimmer, 2019). K_v_1 channels display little/no inactivation, resulting in sustained delayed rectifier K^+^ currents, with the exception of K_v_1.4, which underlies transient A-type K^+^ current.

##### Neurons

K_v_1.1 expression is highest in the brainstem, while K_v_1.4 > K_v_1.2 represent the main K_v_1 subunits in the hippocampus (Trimmer, 2015). K_v_1.1 channels, in association with K_v_1.2, cluster in the juxtaparanodal regions of axons under the myelin sheath and regulate action potential (AP) propagation and neural excitability (Wang et al., 1993; Trimmer and Rhodes, 2004; Ovsepian et al., 2016). Mutations of K_v_1 channels result in hyper-excitability, episodic ataxia, myokymia, and epilepsy (Allen et al., 2020).

##### Glia

Mouse astrocytes express low levels of K_v_1.1, K_v_1.2, and K_v_1.4 transcripts (Smart et al., 1997), but K_v_1.2 and K_v_1.4 expression is high in reactive rat astrocytes (Akhtar et al., 1999). K_v_1.1 transcripts and proteins are highly expressed in C6 glioma cells. Rodent oligodendrocyte precursor cells (OPCs) express K_v_1.1, K_v_1.2, and K_v_1.4 transcripts (Attali et al., 1997; Chittajallu et al., 2002; Falcao et al., 2018; Batiuk et al., 2020) but only K_v_1.4 and low level of K_v_1.2 proteins (Attali et al., 1997; Schmidt et al., 1999). In OPCs and astrocytes, the K_v_1 subunits regulate cell growth and cell cycle progression, e.g., K_v_1.4 overexpression *in vitro* increases OPCs proliferation (Schmidt et al., 1999) while deletion decreases it (Gonzalez-Alvarado et al., 2020). Recent RNA-seq did not detect K_v_1.1, K_v_1.2, and K_v_1.4 in mouse microglia (Hammond et al., 2019), but earlier studies found K_v_1.1 and K_v_1.2 mRNAs and/or proteins in BV2 microglia, rat cultured microglia, and amoeboid microglia within corpus callosum during development, but barely in resting microglia by P21 (Fordyce et al., 2005; Li F. et al., 2008; Wu et al., 2009). In microglia, K_v_1.1 and K_v_1.2 expression was linked to cell activation (Eder, 1998), and their upregulation induced by lipopolysaccharide (LPS), ATP, or hypoxia is involved in the release of pro-inflammatory cytokines and intracellular production of reactive oxygen species (ROS) and nitric oxide (NO) (Li F. et al., 2008; Wu et al., 2009).

##### Expression and Function in MS

Bulk RNA-seq found upregulation of K_v_1.1, K_v_1.2, and Kv1.4 transcripts in CA lesions (Figure 2, Table 1; Elkjaer et al., 2019; Frisch et al., 2020). The snRNA-seq detected significant K_v_1.2 expression in neuronal clusters, slight increase of K_v_1.4 transcripts in neuronal but not glial clusters, and no K_v_1.1 transcript (Tables 1, 2; Jakel et al., 2019).

The CA lesion is characterized by ongoing tissue damage and, functionally, K_v_1.2 upregulation in CA lesions may be a hallmark of axonal damage. While recent data found that *KCNA1* gene is downregulated during demyelination in the cuprizone model (Martin et al., 2018), in animal models of MS, K_v_1.2 (and also K_v_1.1) ectopically redistributes to nodes and internodes of WM axons (McDonald and Sears, 1969; Wang et al., 1995; Sinha et al., 2006; Jukkola et al., 2012; Zoupi et al., 2013; Kastriti et al., 2015), while in human MS, the dislocation of K_v_1.2 channels is associated with paranodal pathology, particularly in NAWM regions, and contributes to axonal dysfunction (Howell et al., 2010; Gallego-Delgado et al., 2020). The upregulated and redistributed K_v_1.2 and K_v_1.1 channels may hyperpolarize the axonal resting membrane potential (V_rest_), elevate the amount of depolarization necessary for AP initiation, and impair AP conduction (Wang et al., 1995; Sinha et al., 2006; Jukkola et al., 2012). Pharmacological inhibition of K_v_1.1 and K_v_1.2 channels, e.g., with 4-aminopyridine, enhances axonal conduction and improves MS symptoms (Lugaresi, 2015).

It is difficult to speculate regarding K_v_1.4 function in MS because data are not consistent. In animal models of MS and spinal cord injury (SCI), this developmentally restricted subunit re-appears/increases in OPCs, OLs, and astrocytic processes around lesion sites (Herrero-Herranz et al., 2007; Jukkola et al., 2012), but not in WM axons or microglia (Edwards et al., 2002; Jukkola et al., 2012). Mice lacking K_v_1.4 exhibit reduced myelin loss in the spinal cord WM during EAE but no change of demyelination/remyelination in the corpus callosum in the cuprizone model (Gonzalez-Alvarado et al., 2020). However, it is unclear whether function of K_v_1.4 subunits is relevant for glial cells in human MS because snRNA-seq barely detected K_v_1.4 transcripts in glia clusters (Table 2).

#### Kv2.1 and Kv2.2 (KCNB1 and KCNB2)

K_v_2 channels (encoded by *KCNB1* and *KCNB2* genes) mediate high-voltage-activated slowly inactivating delayed rectifier K^+^ currents (Guan et al., 2007). K_v_2.1 channels can assemble with electrically silent K_v_S subunits, resulting in greater variability of K_v_2 currents (Trimmer, 2015; Johnson et al., 2019).

##### Neurons

High-density clusters of K_v_2.1 and K_v_2.2 localize to soma, proximal dendrites, and axonal initial segment (AIS). K_v_2 channels influence AP duration during high-frequency firing and regulate neuronal excitability (Guan et al., 2007). K_v_2.1 mutations are associated with neonatal encephalopathy epilepsies and neurodevelopmental delays (Torkamani et al., 2014; Thiffault et al., 2015; de Kovel et al., 2017).

##### Glia

RNA-seq detected *KCNB1* gene in mouse OPC and microglia (Falcao et al., 2018; Hammond et al., 2019).

##### Expression and Function in MS

Bulk RNA-seq revealed upregulation of K_v_2.1 and K_v_2.2 transcripts in CA lesions of SPMS brain (Figure 2, Table 1; Elkjaer et al., 2019; Frisch et al., 2020), while snRNA-seq found K_v_2.1 and K_v_2.2 in neuronal clusters (Tables 1, 2; Jakel et al., 2019). During EAE, K_v_2.1 protein expression was downregulated in spinal cord motor neurons (Jukkola and Gu, 2015). Remarkably, K_v_2.1 channels exist as freely dispersed conducting channels, or form electrically silent somatodendritic clusters (Schulien et al., 2020). Upregulated clustered K_v_2.1 channels promote functional coupling of L-type Ca^2+^ channels in plasma membrane to ryanodine receptors (RyRs) of the endoplasmic reticulum (ER) (Deutsch et al., 2012; Kirmiz et al., 2018; Vierra et al., 2019) and may modulate intracellular Ca^2+^ level contributing to cell damage, while dispersal of K_v_2.1-clusters blocks apoptogenic K^+^ currents and provides neuroprotection (Sesti et al., 2014; Justice et al., 2017). Hence, to elucidate the functional role of K_v_2 upregulation in MS (Table 1), it will be important to determine whether it reflects an increase in clustered or dispersed K_v_2 channels.

#### K_v_3.3 (KCNC3)

The *KCNC3* gene encodes for the K_v_3.3 subunit, which, together with K_v_3.1, K_v_3.2, and K_v_3.4, belongs to the K_v_3 channel subfamily (Shaw). The K_v_3.3 and K_v_3.4 mediate transient A-type K^+^ currents, while K_v_3.1 and K_v_3.2 mediate sustained K^+^ currents.

##### Neurons

K_v_3 channels localize to axonal and somatodendritic domains, and play a critical role in regulating AP firing at high frequency (Rasmussen and Trimmer, 2019). *KCNC3* mutations result in spinocerebellar ataxia type-13 and cerebellar neurodegeneration (Rasmussen and Trimmer, 2019).

##### Glia

Cortical and hippocampal astrocyte cultures express K_v_3.3 and K_v_3.4 mRNAs and proteins (Bekar et al., 2005; Boscia et al., 2017). *KCNC3* mRNA was detected in mouse OPCs and microglia (Larson et al., 2016; Falcao et al., 2018).

##### Expression and Function in MS

Bulk RNA-seq showed significant K_v_3.3 upregulation in CA lesions (Figure 2, Table 1), while snRNA-seq revealed its predominant distribution in neuronal clusters (Table 2; Jakel et al., 2019). K_v_3.3 may play a detrimental role in MS because it increases in injured WM axons during EAE progression in mice and in human MS lesions (Jukkola et al., 2017), and the deletion of K_v_3.1, which forms hetero-tetramers with K_v_3.3, reduced EAE severity in mice (Jukkola et al., 2017).

#### Kv4.2 (KCND2)

The *KCND2* gene encodes for the K_v_4.2 subunit that (together with K_v_4.1 and K_v_4.3) is a member of the K_v_4 channel subfamily (Shal) and is highly expressed in the brain (Alfaro-Ruiz et al., 2019). K_v_4 channels activate at subthreshold potentials and then inactivate and recover rapidly. They mediate transient A-type K^+^ current (Bahring et al., 2001; Birnbaum et al., 2004).

##### Neurons

K_v_4.2 subunits are highly expressed in soma and dendrites of hippocampal neurons and interneurons. They regulate the threshold for AP initiation and repolarization, frequency-dependent AP broadening, and AP back-propagation (Nerbonne et al., 2008). K_v_4.2 mutations are associated with infant-onset epilepsy and autism.

##### Glia

K_v_4.2-transcripts were found in mouse astrocytes (Bekar et al., 2005) and OPCs, but only at very low levels in microglia (Falcao et al., 2018; Hammond et al., 2019; Batiuk et al., 2020).

##### Expression and Function in MS

Bulk RNA-seq found significant K_v_4.2 upregulation in CA lesions (Figure 2, Table 1; Elkjaer et al., 2019; Frisch et al., 2020). The snRNA-seq reported significant expression of K_v_4.2 transcripts in neuronal, OPCs, and committed OPCs (COP) clusters (Table 2; Jakel et al., 2019). K_v_4.2 subunit may contribute to oligodendrocyte dysfunction in SPMS brain because dysregulated *KCND2* transcripts are associated with oligodendrocyte dysfunction in mental illnesses (Vasistha et al., 2019).

#### K_v_7.2, K_v_7.3, and K_v_7.5 (KCNQ2, KCNQ3, and KCNQ5)

The *KCNQ* genes encode for K_v_7.1–K_v_7.5 (*KCNQ1–KCNQ5*) family members that underlie a voltage-gated non-inactivating outward K^+^ current, known as M current (I_M_).

##### Neurons

The K_v_7.2/3 or K_v_7.3/5 hetero-tetramers represent the dominant subunit composition in neurons (Wang et al., 1998; Cooper et al., 2000; Kharkovets et al., 2000), while K_v_7.4/K_v_7.5 is dominant in vascular smooth muscles (Brueggemann et al., 2014). The K_v_7.2- and K_v_7.3 subunits co-cluster with Na_v_ channels at AIS and nodes of Ranvier in rodent somatosensory cortex and spinal cord WM and gray matter (GM) (Pan et al., 2006; Cooper, 2011; Battefeld et al., 2014). K_v_7.5 localizes to soma and dendrites of cortical and hippocampal neurons and contributes to afterhyperpolarization currents (Tzingounis et al., 2010). The K_v_7 channels stabilize V_rest_, influence neuronal subthreshold excitability, and regulate spike generation (Jentsch, 2000; Miceli et al., 2008). By reducing the steady-state inactivation of nodal Na_v_ channels, the K_v_7 channels increase the availability of transient Na_v_ currents at nodes of Ranvier, thereby accelerating the AP upstroke and elevating short-term axonal excitability (Hamada and Kole, 2015). In the perisomatic region, K_v_7 channels counteract the persistent Na_v_ current and restrain repetitive firing (Pan et al., 2006; Cooper, 2011). Variants of *KCNQ2/KCNQ3* or *KCNQ4* genes cause developmental/epileptic disorders and hearing loss (Soldovieri et al., 2011; Miceli et al., 2013).

##### Glia

*KCNQ3* gene is expressed in spinal cord WM astrocytes (Devaux et al., 2004), while *KCNQ5* is expressed in rat retinal astrocytes (Caminos et al., 2015). The *KCNQ2-5* mRNAs and proteins were detected in rat cortical OPCs and microglia cultures, while differentiated oligodendrocytes showed weak *KCNQ4* expression (Wang et al., 2011; Vay et al., 2020).

##### Expression and Function in MS

Bulk RNA-seq found upregulation of *KCNQ2-3-5* transcripts in CA lesions (Figure 2, Table 1; Elkjaer et al., 2019; Frisch et al., 2020). The snRNA-seq reported *KCNQ2-3-5* expression in neuronal clusters and *KCNQ3* expression in immune oligodendroglia (ImOLG) and microglia/macrophages clusters (Tables 1, 2; Jakel et al., 2019). K_v_7.3 upregulation may reflect increased necessity of the channels along the axons because K_v_7.3 subunit extensively redistributes to internodes of acutely and chronically demyelinated GM axons in the cuprizone model (Hamada and Kole, 2015). It is tempting to speculate that K_v_7 upregulation may be beneficial during MS. First, K_v_7 channels may increase the availability of transient Na_v_ current *via* membrane hyperpolarization supporting AP conduction in demyelinated axons (Battefeld et al., 2014). Second, K_v_7 channels may mitigate inflammation-induced neuronal excitability because, following LPS exposure, the I_M_ inhibition underlies hyperexcitability of hippocampal neurons that is reversed by a nonselective K_v_7-opener retigabine (Tzour et al., 2017). Although retigabine also exerts neuroprotective effects in several neurodegenerative conditions (Boscia et al., 2006; Nodera et al., 2011; Wainger et al., 2014; Bierbower et al., 2015; Li et al., 2019; Vigil et al., 2020; Wu et al., 2020), a clinical trial with retigabine analog flupirtine failed to demonstrate neuroprotective effects during MS (Dorr et al., 2018). Furthermore, blockade of K_v_7 channels with XE-991 inhibited migration of LPS-treated pro-inflammatory microglia *in vitro* (Vay et al., 2020), suggesting that these channels may promote the pro-inflammatory role of microglia also during MS. Hence, neuronal and glial K_v_7 channels may have diverse functions during MS.

#### K_v_8.1 and K_v_9.2 (KCNV1 and KCNS2)

##### Neurons

*KCNV1* and *KCNS2* genes encode for electrically silent (K_v_S) K_v_8.1- and K_v_9.2 subunits that assemble into hetero-tetrameric channels with K_v_2 subunits (Bocksteins, 2016). A number of channelopathies is ascribed to K_v_S subunits (Salinas et al., 1997a; Liu et al., 2016; Allen et al., 2020), pointing to their important physiological role.

##### Glia

*KCNV1* and *KCNS2* transcripts were found in oligodendrocyte lineage cell (Marques et al., 2016).

##### Expression and Function in MS

Bulk RNA-seq showed upregulation of *KCNV1* and *KCNS2* genes in CA lesions (Figure 2, Table 1; Elkjaer et al., 2019; Frisch et al., 2020). The snRNA-seq detected *KCNV1* and *KCNS2* in neuronal clusters (Table 2; Jakel et al., 2019). Co-assembly between K_v_8.1 and K_v_2.1 reduces K_v_2.1 current density (Hugnot et al., 1996; Castellano et al., 1997): the high stoichiometry of the K_v_8.1 subunit suppresses surface expression and favors retention of heteromeric channels in the ER (Salinas et al., 1997b). Neurons with reduced K_v_2.1-mediated currents demonstrate broadened APs (Du et al., 2000) underlying hyper-synchronized high-frequency firing observed during epilepsy. Hence, upregulated K_v_S subunits in CA lesions may influence the localization of clustered K_v_2 subunits in SPMS brain and affect AP firing and/or propagation.

#### Eag2, erg3, and elk1 (KCNH5, KCNH7, and KCNH8)

*KCNH* genes encode for K_v_10–K_v_12 subfamilies, all orthologs of the Drosophila ether-àgo-go (EAG) channels. They include two eag (K_v_10), three eag-related (erg/K_*v*_11), and three eag-like (elk/K_v_12) K^+^ channels that can form heteromeric channels within each subfamily (Rasmussen and Trimmer, 2019).

##### Neurons

All EAG channels are expressed in the CNS neurons (Ludwig et al., 2000; Papa et al., 2003; Zou et al., 2003), but only erg-mediated currents have been verified using suitable blockers (Bauer and Schwarz, 2018).

##### Glia

RNA-seq detected *KCNH5, KCNH7*, and *KCNH8* expression in mouse OPCs (Falcao et al., 2018). *KCNH5* and *KCNH7* genes were found in astrocytes (Batiuk et al., 2020), while only the KCNH7 gene was detected in mouse microglia (Hammond et al., 2019). Erg-type currents were reported in neopallial microglia cultures (Zhou et al., 1998) and hippocampal astrocytes (Emmi et al., 2000; Papa et al., 2003).

##### Expression and Function in MS

Bulk RNA-seq detected increased *KCNH5(eag2)* and *KCNH7(erg3)* transcripts in CA lesions and downregulation of *KCNH8(elk1)* transcript in all lesions and NAWM (Figure 2, Table 1; Elkjaer et al., 2019; Frisch et al., 2020). The snRNA-seq found significant expression of *KCNH5* and *KCNH7* transcripts in neuronal clusters and *KCNH8* in mature oligodendrocyte clusters (Tables 1, 2; Jakel et al., 2019). The functional role of *eag2, erg3*, and *elk1* during MS may be related to altered neuronal excitability. Indeed, human *eag1* and *eag2* gain-of-function mutations underlie severe neurological disorders associated with epileptic seizures (Allen et al., 2020). The *erg* channels that are active at subthreshold potentials stabilize the V_rest_ and dampen excitability (Fano et al., 2012). *Erg3* knockdown in mice increases intrinsic neuronal excitability and enhances seizure susceptibility, while treatment with *erg* activator reduces epileptogenesis (Xiao et al., 2018). *Erg3* expression is decreased in the brain of epilepsy patients. Remarkably, association of *KCNH7(erg)* intronic polymorphisms with MS pathogenesis was speculated although never substantiated (Martinez et al., 2008; Couturier et al., 2009).

### Two-Pore Domain K^+^ Channels (K2P)

K2P K^+^ channels are encoded by 15 *KCNK* genes, stratified into six subfamilies: TWIK, TASK (TWIK-related acid-sensitive), TREK (TWIK-related arachidonic acid activated), THIK (tandem pore domain halothane-inhibited), TALK (TWIK-related alkaline pH-activated), and TRESK (TWIK-related spinal cord) K^+^ channels (Enyedi and Czirjak, 2010). K2P K^+^ channels contribute to “leak” K^+^ current, helping to establish and maintain V_rest_ (Enyedi and Czirjak, 2010).

#### TREK1 and TREK2 (KCNK2 and KCNK10)

##### Neurons and Glia

*KCNK2* and *KCNK10* genes encode for TREK-1 and TREK-2 channels, which are expressed in neurons, astrocytes, and OPC (Hervieu et al., 2001; Talley et al., 2001; Falcao et al., 2018). Only TREK-1 transcripts were detected in microglia (Hammond et al., 2019). In astrocytes, TREK channels contribute to passive conductance and glutamate release (Zhou et al., 2009; Woo et al., 2012). TREK-1 and TREK-2 may be activated by a wide range of physiological and pathological stimuli reminiscent of inflammatory environment including membrane stretch, heat, intracellular acidosis, and cellular lipids (Ehling et al., 2015).

##### Expression and Function in MS

Bulk RNA-seq found upregulated TREK-1 transcripts in CA lesions, but a divergent modulation was observed for TREK-2 mRNAs in ALs (Figure 2, Tables 1, 2; Elkjaer et al., 2019; Frisch et al., 2020). *KCNK2* and *KCNK10* transcripts were detected in neuronal and oligodendrocyte clusters, but scarcely observed in astrocytes (Table 2; Jakel et al., 2019). TREK-1 upregulation in CA lesions most likely reflects a protective response because TREK-1 plays a neuroprotective role during neurological diseases, including MS (Djillani et al., 2019). TREK-1 reduces neuronal excitability by hyperpolarizing the membrane potential (Honore, 2007) and is required for rapid AP repolarization at the node of Ranvier in mammalian afferent myelinated nerves, while TREK-1 loss-of-function retards nerve conduction and impairs sensory responses in animals (Kanda et al., 2019). Treatment of mice with TREK-1 activators, riluzole (Gilgun-Sherki et al., 2003), or alpha-linolenic acid attenuates EAE course (Blondeau et al., 2007), while these effects are reduced in TREK-1^−/−^ mice (Bittner et al., 2014). TREK-1 function is also important for non-neuronal cells because aggravated EAE course in TREK-1^−/−^ mice is associated with increased numbers of infiltrating T cells and higher endothelial expression of ICAM1 and VCAM1 (Bittner et al., 2013), and TREK-1 is reduced in the microvascular endothelium in inflammatory MS brain lesions (Bittner et al., 2013).

TREK-2 downregulation in AL, a lesion type characterized by myelin breakdown and infiltration by inflammatory cells (Elkjaer et al., 2019; Frisch et al., 2020), may contribute to reduced glutamate and K^+^ buffering and neuronal over-excitation because TREK-2 helps maintain the membrane potential and low extracellular glutamate and K^+^ level during ischemia (Gnatenco et al., 2002; Rivera-Pagan et al., 2015).

### Na^+^- and Ca^2+^-Activated K^+^ Channels

#### K_Na_1.1 (KCNT1)

##### Neurons

The *KCNT1* and *KCNT2* genes encode for Slack and Slick K^+^ channels that are activated by Na^+^ influx (Bhattacharjee and Kaczmarek, 2005). They localize to soma and axons of neurons (Bhattacharjee et al., 2002; Brown et al., 2008; Rizzi et al., 2016) and are involved in the generation of slow after-hyperpolarization, regulation of firing patterns, and setting and stabilizing the V_rest_ (Franceschetti et al., 2003). Alterations in *KCNT1* and *KCNT2* genes are linked to early-onset epileptic encephalopathies and Fragile-X-syndrome (Kim and Kaczmarek, 2014).

##### Glia

RNA-seq detected *KCNT1* gene in mouse astrocytes (Batiuk et al., 2020).

##### Expression and Function in MS

Bulk RNA-seq showed K_Na_1.1 upregulation in CA lesions (Figure 2, Table 1; Elkjaer et al., 2019; Frisch et al., 2020). SnRNA-seq detected *KCNT1* in neuronal clusters (Table 2). *KCNT1* function in MS may be related to myelination/demyelination because severely delayed myelination occurs in patients with *KCNT1* mutations (Vanderver et al., 2014). Furthermore, *KCNT1* is a causative gene in infants with hypomyelinating leukodystrophy showing WM alterations (Arai-Ichinoi et al., 2016), and *KCNT1* mutations occur in infant epilepsy associated with delayed myelination, thin corpus callosum, and WM hyper-intensity in MRI (McTague et al., 2013; Shang et al., 2016; Borlot et al., 2020).

#### K_Ca_2.3, SK3 (KCNN3)

The *KCNN3* gene encodes for the SK3 subunit of small-conductance Ca^2+^-activated K^+^ channels (SK channels). They mediate Ca^2+^ gated K^+^ current and thus couple the increase in intracellular Ca^2+^ concentration to hyperpolarization of the membrane potential.

##### Neurons

SK3 channels are found on dendrites and AIS (Abiraman et al., 2018). They play a role in AP propagation and regulation of neuronal excitability (Stocker, 2004). They protect against excitotoxicity by maintaining Ca^2+^ homeostasis after NMDA receptor activation (Dolga et al., 2011).

##### Glia

RNA-seq detected intense *KCNN3* expression in mouse astrocytes (Batiuk et al., 2020), confirming earlier studies, which showed labeling of GFAP^+^ processes in the supraoptic nucleus for SK3 channels and suggested the role of SK3 in astrocytic K^+^ buffering (Armstrong et al., 2005). Oligodendrocyte lineage cells express low levels of *KCNN3* mRNA (Falcao et al., 2018), while mouse microglia does not express *KCNN3* (Hammond et al., 2019). However, rat microglia in culture expresses the SK3 subunit, which is increased upon microglia activation with LPS (Schlichter et al., 2010). SK3 activation inhibited microglia proliferation, inflammatory IL-6 production, and morphological transformation to macrophages, while blocking SK3 in microglia-reduced neurotoxicity (Dolga et al., 2012).

##### Expression and Function in MS

Bulk RNA-seq showed significant and unique downregulation of *KCNN3* in ILs (Elkjaer et al., 2019; Frisch et al., 2020). SnRNA-seq revealed high *KCNN3* expression in astrocyte clusters (Figure 2, Table 1; Jakel et al., 2019). ILs consist of large demyelinated areas devoid of macrophages but filled with scar-forming astrocytes showing reduced ability to buffer glutamate and K^+^ (Compston and Coles, 2008; Kuhlmann et al., 2017; Filippi et al., 2018; Schirmer et al., 2018). Hence, *KCNN3* downregulation in MS may reflect altered function of astrocytes, e.g., K^+^ buffering (Armstrong et al., 2005), contributing to axonal hyper-excitability and death.

### Inward Rectifier K^+^ Channels (K_ir_)

*KCNJ* gene family encodes K_ir_ channels and comprises 16 subunits of K_ir_1–K_ir_7 subfamilies categorized into four groups: (1) classical (K_ir_2.x); (2) G-protein-gated (K_ir_3.x); (3) ATP-sensitive (K_ir_6.x); and (4) K^+^-transport channels (K_ir_1.x, K_ir_4.x, K_ir_5.x, K_ir_7.x) (Hibino et al., 2010). At a comparable driving force, K_ir_ channels allow greater influx than efflux of K^+^-ions. Their high open probability at negative transmembrane voltages makes them well-suited to set the V_rest_ and to control cell excitability.

#### Kir3.2 (KCNJ6)

*KCNJ6* gene encodes for K_ir_3.2 subunits, also known as G-protein-gated K_ir_ (GIRK2) channels that are effectors for G_i_/_o_-dependent signaling and mediate outward K^+^ current.

##### Neurons

K_ir_3.1/K_ir_3.2 hetero-tetramers are found in the somatodendritic compartment of neurons. Activation of GIRK channels is mediated by G-protein-coupled receptors including muscarinic, metabotropic glutamate, somatostatin, dopamine, endorphins, endocannabinoids, etc. GIRK channels are important for K^+^ homeostasis and maintenance of V_rest_ near the K^+^ equilibrium potential. GIRK current hyperpolarizes neuronal membrane reducing spontaneous AP firing and inhibiting neurotransmitter release (Luscher and Slesinger, 2010). GIRK signaling contributes to learning/memory, reward, pain, anxiety, schizophrenia, addiction, and other processes (Mayfield et al., 2015). K_ir_3.2 mutations in mice lead to a loss of K^+^ selectivity and increased Na^+^ permeability of the channel, resulting in the *weaver* phenotype (Liao et al., 1996; Surmeier et al., 1996).

##### Glia

Astrocytes and Müller cells express K_ir_3 channels (Raap et al., 2002). K_ir_3.2 transcripts were detected in the mouse optic nerve (Papanikolaou et al., 2020) and oligodendrocyte lineage (Falcao et al., 2018), but not in microglia (Hammond et al., 2019).

##### Expression and Function in MS

RNA-seq revealed *KCNJ6* upregulation in the CA lesions (Elkjaer et al., 2019; Frisch et al., 2020). The snRNA-seq predominantly found *KCNJ6* transcripts in neuronal clusters (Jakel et al., 2019; Table 1). The functional role of K_ir_3.2 channels in MS may be related to membrane hyperpolarization and compensation of excessive neuronal excitability driving neurodegeneration.

#### Kir5.1 (KCNJ16)

*KCNJ16* gene encodes for K_ir_5.1 subunit, which forms an electrically silent channel when combined with K_ir_2.1 (Derst et al., 2001; Pessia et al., 2001), but is functional when combined with K_ir_4.1 (Konstas et al., 2003). Clustering of heteromeric K_ir_4.1/K_ir_5.1 and homomeric K_ir_5.1 channels on plasmalemma involves the anchoring protein PSD-95 (Tanemoto et al., 2002; Brasko et al., 2017). Heteromeric K_ir_4.1/K_ir_5.1 channels exhibit larger channel conductance, greater pH sensitivity, and different expression patterns if compared to K_ir_4.1 homomers (Tanemoto et al., 2000; Tucker et al., 2000; Pessia et al., 2001; Hibino et al., 2010).

##### Neurons

In cultures, K_ir_5.1 immunoreactivity was detected in somatodendritic compartments where PSD-95 immunoreactivity was also localized. The K_ir_5.1/PSD-95 complex may exist at dendritic spines *in vivo* and play a role in synaptic transmission (Tanemoto et al., 2002).

##### Glia

K_ir_5.1 mRNA is two-fold higher in OPCs (NG2^+^-glia) vs. astrocytes (Zhang et al., 2014), and mouse brain microglia expresses K_ir_5.1 transcript too (Hammond et al., 2019). K_ir_5.1 expression in oligodendrocytes and astrocytes depends on its association with K_ir_4.1: loss of K_ir_4.1 reduces K_ir_5.1, suggesting that altered expression/distribution of K_ir_5.1 may contribute to the phenotype of K_ir_4.1 knockout mice (Brasko et al., 2017; Schirmer et al., 2018). The oligodendroglial K_ir_5.1/K_ir_4.1 channels are important for K^+^ clearance (Poopalasundaram et al., 2000; Neusch et al., 2001), long-term maintenance of axonal function, and WM integrity (Kelley et al., 2018; Schirmer et al., 2018). In astrocytes, K_ir_5.1/K_ir_4.1 channels contribute to chemoreception, spatial K^+^ buffering, and breathing control (Mulkey and Wenker, 2011).

##### Expression and Function in MS

Bulk RNA-seq revealed K_ir_5.1 upregulation in CA lesions (Table 1; Elkjaer et al., 2019; Frisch et al., 2020). SnRNA-seq detected K_ir_5.1 in OPCs clusters and scarcely in astrocytes. The *KCNJ16* gene is upregulated during demyelination and acute remyelination in mouse cuprizone model (Martin et al., 2018). Upregulation of K_ir_5.1 may reflect the role of the oligodendroglial K_ir_4.1/K_ir_5.1 channels in K^+^ clearance during MS and may represent a mechanism to compensate K_ir_4.1 reduction in MS brain (Schirmer et al., 2014). Alternatively, K_ir_5.1 upregulation may underlie reduced K_ir_4.1 function in MS because presence of K_ir_5.1 subunit confers loss of functional activity to K_ir_4.1/K_ir_5.1 channels under oxidative stress (Jin et al., 2012).

### Voltage-Gated Na^+^ Channels (Na_v_)

In the mammalian brain, Na_v_ are composed of α-subunit (260 kDa) and one or several β-subunits (β1–β4, of 33–36 kDa) (Goldin et al., 2000). The α-subunit forms the channel pore and acts as a voltage sensor; β-subunits play a modulatory role and influence voltage dependence, gating kinetics, and surface expression of the channel (Goldin et al., 2000; Yu and Catterall, 2003; Namadurai et al., 2015). The nine Na_V_1.1–Na_V_1.9 α-subunits are encoded by the corresponding genes *SCN1A*–*SCN5A* and *SCN8A*–*SCN11A*. In addition, Na_X_ isoform was described,which is encoded by the *SCN6/7A* gene.

#### Na_V_1.1 (SCN1A)

##### Neurons

Na_V_1.1 channels localize to the somatodendritic compartment of principal neurons and AIS of GABAergic interneurons, spinal cord motor neurons, and retinal neurons (Ogiwara et al., 2007; Duflocq et al., 2008; Dumenieu et al., 2017). Na_V_1.1 channels are also present at the nodes of Ranvier of the cerebellar WM, fimbria, corpus callosum, and spinal cord WM (Ogiwara et al., 2007; Duflocq et al., 2008; O'Malley et al., 2009). They play a role during saltatory conduction along myelinated axons and are essential for maintaining the sustained firing of GABAergic interneurons and Purkinje cells, thus controlling the excitability of neuronal networks (Duflocq et al., 2008; Dumenieu et al., 2017). Mutations in Na_V_1.1 channels result in various types of epilepsy and reduced volume of brain GM and WM (Lee et al., 2017; Scheffer and Nabbout, 2019).

##### Glia

Human astrocytes show negligible immunolabelling for Na_V_1.1 and no upregulation in the WM of MS patients (Black et al., 2010). Transcriptome analysis revealed low level of SCN1A in mouse cortical and hippocampal astrocytes (Batiuk et al., 2020). RNA-seq detected *SCN1A* in oligodendrocytes and OPCs throughout the CNS (Larson et al., 2016; Marques et al., 2016; Falcao et al., 2018). The functional role of Na_V_1.1 channels in astrocytes and oligodendroglia remains unknown. Transcriptome studies have not detected *SCN1A* in microglia prepared from brain homogenates (Hammond et al., 2019), but Na_V_1.1 protein was found in microglia derived from neonatal rat mixed glial cultures (Black et al., 2009). Na_V_1.1 channels may be involved in regulation of phagocytosis and/or release of IL-1α, IL-β, and TNF-α from microglia (Black et al., 2009). The Na_V_1.1 mRNA was detected in astrocytoma, oligodendroglioma, and glioblastoma samples from patients where these channels may contribute to the pathophysiology of brain tumors (Schrey et al., 2002).

##### Expression and Function in MS

Bulk RNA-seq detected *SCN1A* upregulation in CA lesions (Figure 2, Table 1; Elkjaer et al., 2019; Frisch et al., 2020). The snRNA-seq revealed significant expression of Na_V_1.1 transcripts in neuronal, committed OPC, and OPC clusters (Tables 1, 2; Jakel et al., 2019). Experimental models do not provide clues regarding the functional role of Na_V_1.1 channels in MS: Na_V_1.1 expression was increased or unaltered in the optic nerve during EAE (Craner et al., 2003; O'Malley et al., 2009), while in the spinal cord, these channels clustered at the nodes of Ranvier and localized along the demyelinated regions (O'Malley et al., 2009). *SCN1A* upregulation in human MS may reflect the necessity of the channel for redistribution along the demyelinated axons and support of AP propagation.

#### Na_V_1.2 (SCN2A)

##### Neurons

The Na_V_1.2 channels localize to the AIS, immature nodes of Ranvier, and in non-myelinated axons during early development. As nervous system matures, Na_V_1.2 channels are replaced by Na_V_1.6 channels (Boiko et al., 2001; Osorio et al., 2005; Dumenieu et al., 2017), although in some neurons, they remain into adulthood. Na_v_1.2 channels of the AIS control back-propagation of APs into the somatodendritic compartment, while Na_V_1.6 channels are being placed at distal parts of the AIS control initiation and propagation of AP into the axon (Boiko et al., 2003; Hu et al., 2009). Na_V_1.2 channels are also diffusely distributed along non-myelinated axons in the adult CNS where they may support slow spike propagation (Arroyo et al., 2002; Dumenieu et al., 2017).

##### Glia

Na_V_1.2 protein was found in rat astrocytes isolated from the spinal cord and optic nerve (Black et al., 1995), but only limited Na_V_1.2 expression was observed in human astrocytes in control and MS tissue (Black et al., 2010). The RNA-seq detected *SCN2A* expression in oligodendrocytes and OPCs (Larson et al., 2016; Marques et al., 2016). Knockdown of Na_V_1.2 in pre-oligodendrocytes of the auditory brainstem resulted in reduced number and length of cellular processes and decreased MBP level, indicating that Na_V_1.2 channels are important for structural maturation of myelinating cells and myelination (Berret et al., 2017). Microglia expresses no/little functional Na_V_1.2 channels (Black et al., 2009; Pappalardo et al., 2016; Hammond et al., 2019).

##### Expression and Function in MS

Bulk RNA-seq detected upregulation of *SCN2A* gene in CA lesions (Figure 2, Table 1; Elkjaer et al., 2019; Frisch et al., 2020), while snRNA-seq showed abundant *SCN2A* expression in neuronal clusters (Tables 1, 2; Jakel et al., 2019). The upregulation may reflect re-expression of Na_V_1.2 protein, in line with previous reports showing diffuse distribution of Na_V_1.2 channels along the demyelinated axons in human MS lesions within optic nerve and spinal cord (Craner et al., 2004b). Axonal Na_V_1.2 channels may contribute to preservation of AP propagation and re-establishment of myelin sheathes (Coman et al., 2006), as it occurs during development. On the other hand, Na_V_1.2 channels may promote axonal damage by increasing the intracellular Na^+^ concentration that triggers reversal of Na^+^/Ca^2+^ exchanger (NCX) and Ca^2+^ overload in the axoplasm (Friese et al., 2014; Schattling et al., 2016). In line with this, human gain-of-function mutation in the mouse *SCN2A* gene triggers axonal damage, neurodegeneration, disability, and lethality in the mouse model of MS (Schattling et al., 2016). Expression of “developmental” Na_V_1.2 channels in axons was also found in animal models of MS, i.e., in adult *Shiverer* mice that lack myelin (Westenbroek et al., 1992; Boiko et al., 2001), in transgenic mice overexpressing proteolipid protein that initially have normal myelination but then lose myelin (Rasband et al., 2003), and in the demyelinated optic nerve and spinal cord during EAE (Craner et al., 2003, 2004a; Herrero-Herranz et al., 2008). However, other data showed that in chronic spinal cord MS lesions, Na_V_1.2 channels localize on astrocytic processes surrounding the axons rather than on axons themselves (Black et al., 2007), and Na_V_1.2 expression/distribution was unchanged in the spinal cord of myelin-deficient rats (Arroyo et al., 2002).

#### Na_V_1.3 (SCN3A)

##### Neurons

Na_V_1.3 channels are highly expressed in rodent and human CNS throughout the embryonic development (Black and Waxman, 2013). Some studies reported that their expression decreases during the first weeks after birth, while others found Na_V_1.3 immunoreactivity in GM and/or WM of adult rat and human brain (Whitaker et al., 2001; Lindia and Abbadie, 2003; Thimmapaya et al., 2005; Cheah et al., 2013). Na_V_1.3 channels mainly localize to the somatodendritic compartment of neurons but were also detected along the axons including myelinated fibers where they may contribute to initiation and propagation of APs (Whitaker et al., 2001; Lindia and Abbadie, 2003; Cheah et al., 2013; Wang et al., 2017). In the developing brain, Na_V_1.3 channels regulate proliferation and migration of cortical progenitors that do not fire APs (Smith et al., 2018).

##### Glia

The mRNA and Na_V_1.3 protein were detected in astrocytes (Black et al., 1995). RNA-seq demonstrated *SCN3A* expression in oligodendroglial cells and suggested higher expression in OPCs vs. mature oligodendrocytes (Larson et al., 2016; Marques et al., 2016). Na_V_1.3 expression in microglia was negligible or absent (Black et al., 2009; Hammond et al., 2019). Heterogeneous expression (from weak to strong) of Na_V_1.3 mRNA occurred in human astrocytoma, oligodendroglial tumors, and glioblastoma (Schrey et al., 2002). Functions of Na_V_1.3 channels in glia remain unknown.

##### Expression and Function in MS

Bulk mRNA-seq reported upregulation of *SCN3A* gene in the CA lesions (Elkjaer et al., 2019; Frisch et al., 2020). The snRNA-seq found significant *SCN3A* expression in neuronal and OPCs clusters (Jakel et al., 2019; Tables 1, 2). *SCN3A* upregulation during MS may reflect augmented expression of Na_V_1.3 protein in axons that is necessary for supporting/re-establishment of AP propagation in injured WM, because increased Na_V_1.3 levels are known to be associated with higher neuronal firing. For instance, mRNA and Na_V_1.3 protein were upregulated in spontaneously epileptic rats (Guo et al., 2008), and expression in hippocampal neurons of a novel coding variant SCN3A-K354Q resulted in enhanced Na_v_1.3 currents, spontaneous firing, and paroxysmal depolarizing shift-like depolarizations of the membrane potential (Estacion et al., 2010).

#### Na_V_1.6 (SCN8A)

##### Neurons

Na_V_1.6 channels cluster at high-density at the AIS and nodes of Ranvier of GM and WM axons, but can be also located on the soma, dendrites, and synapses although at a lower density (Caldwell et al., 2000; Dumenieu et al., 2017; Johnson et al., 2017; Eshed-Eisenbach and Peles, 2020). The expression level of Na_V_1.6 channels is low during development, but significantly increases as the nervous system matures (Boiko et al., 2001; Osorio et al., 2005; Dumenieu et al., 2017). In the adult CNS, Na_V_1.6 channels are the major Na^+^ channels responsible for initiation and propagation of APs (Boiko et al., 2003; Hu et al., 2009). Loss of Na_v_1.6 activity results in decreased neuronal excitability, while gain-of-function mutations potentiate excitability (O'Brien and Meisler, 2013). *SCN8A* mutations in mice result in ataxia, tremor, and dystonia; in humans, *SCN8A* haploinsufficiency is associated with intellectual disability, while hyperactivity can contribute to pathogenesis of epileptic encephalopathy (O'Brien and Meisler, 2013; Meisler, 2019).

##### Glia

RNA-seq detected *SCN8A* transcripts in mouse oligodendrocyte lineage (Marques et al., 2016), but they were negligible in microglia (Hammond et al., 2019). Immunoreactivity for Na_V_1.6 was observed in cultured spinal cord astrocytes and in brain microglia *in vitro* and *in situ* (Reese and Caldwell, 1999; Black et al., 2009; Black and Waxman, 2012; Hossain et al., 2013), but their functional role is unknown.

##### Expression and Function in MS

Bulk mRNA-seq found upregulation of *SCN8A* gene in CA lesions (Elkjaer et al., 2019; Frisch et al., 2020), while snRNA-seq did not detect *SCN8A* transcripts (Jakel et al., 2019) (Tables 1, 2). Upregulation of *SCN8A* may reflect increased diffuse distribution of the channels along the demyelinated axons; it may be important for remyelination but may also contribute to axonal damage. Re-distribution of Na_V_1.6 channels, in parallel to their loss from the nodes of Ranvier, was reported previously in chronic, active, and inactive MS plaques within cerebral hemisphere, cerebellum, and spinal cord WM tissue from MS patients (Craner et al., 2004b; Black et al., 2007; Howell et al., 2010; Bouafia et al., 2014), as well as in several CNS regions affected by demyelination in animal models, including optic nerve and spinal cord WM (Craner et al., 2003, 2004a,b; Hassen et al., 2008; Howell et al., 2010). Expression of Na_V_1.6 channels is disrupted at the nodes of Ranvier of WM axons in Shiverer mice that lack compact myelin (Boiko et al., 2001, 2003), and in transgenic mice overexpressing proteolipid protein that initially have normal myelination but then lose myelin (Rasband et al., 2003). During EAE in animals, Na_V_1.6 co-localizes with NCX and may contribute to persistent Na^+^ influx, increased Na^+^ level in the axoplasm, reversal of NCX, and intra-axonal Ca^2+^ overload leading to axonal damage (Craner et al., 2004a). Interestingly, robust increase in Na_V_1.6 expression was detected also in microglia/macrophages and was associated with microglia activation and phagocytosis in human MS brain and in the EAE model (Craner et al., 2005). *SCN8A* deletion resulted in reduced inflammation and improved axonal health during EAE (Alrashdi et al., 2019). Hence, microglial Na_V_1.6 may contribute to the pathophysiology of MS as well, yet, snRNA-seq did not detect *SCN8A* in WM glia clusters (Tables 1, 2).

#### Na_V_1.9 (SCN11A)

##### Neurons

Although Na_V_1.9 channels are mainly expressed in sensory ganglia neurons (Wang et al., 2017), Na_V_1.9 mRNA and/or protein were detected in soma and/or proximal processes of neurons in the olfactory bulb, hippocampus, cerebellar cortex, supraoptic nucleus, and spinal cord of rodents and humans (Jeong et al., 2000; Blum et al., 2002; Subramanian et al., 2012; Wetzel et al., 2013; Black et al., 2014; Kurowski et al., 2015). Information regarding axonal labeling for Na_V_1.9 is lacking. Na_V_1.9 channels regulate excitation in hippocampal neurons in concert with BDNF and TrkB, control activity-dependent axonal elongation in spinal cord motoneurons, and mediate sustained depolarizing current upon activation of M1 muscarinic receptors in cortical neurons (Blum et al., 2002; Subramanian et al., 2012; Kurowski et al., 2015). It is uncertain whether, similar to their role in the PNS (Cummins et al., 1999; Wang et al., 2017), Na_V_1.9 channels contribute to the regulation of V_rest_ and AP threshold in the CNS neurons.

##### Glia

Very little expression of Na_V_1.9 channels occurs in astrocytes, myelinating glia, and microglia (Marques et al., 2016; Pappalardo et al., 2016).

##### Expression and Function in MS

Bulk mRNA-seq showed *SCN11A* downregulation in ILs (Elkjaer et al., 2019; Frisch et al., 2020). By contrast, snRNA-seq did not detect *SCN11A* mRNA (Jakel et al., 2019; Tables 1, 2). Functional consequence of *SCN11A* downregulation in MS is unknown.

### Voltage-Gated Ca^2+^ Channels (VGCCs)

The VGCCs are composed of α1-, β-, α2/δ-, and γ-subunits (Catterall, 2011; Zamponi et al., 2015). The pore-forming α1-subunit determines channel activity, whereas other subunits are auxiliary and regulate function of α1-subunit. In mammalian cells, 10 different α1-subunits, encoded by different genes, classify into three subfamilies: Ca_V_1, Ca_V_2, and Ca_V_3 (Catterall, 2011; Zamponi et al., 2015; Alves et al., 2019). Depending on the pharmacological properties and activation voltage of Ca^2+^ currents, five different types of VGCCs are distinguished: L-type, N-type, P/Q-type, R-type, and T-type.

### L-Type VGCCs

The α1-subunit of L-type VGCCs is encoded by *CACNA1S* (Ca_V_1.1), *CACNA1C* (Ca_V_1.2), *CACNA1D* (Ca_V_1.3), or *CACNA1F* (Ca_V_1.4) genes. High sensitivity to dihydropyridine modulators distinguishes L-type Ca^2+^ channels from other types of VGCCs. In the CNS, mainly Ca_V_1.2 and Ca_V_1.3 subunits are expressed (Lipscombe et al., 2004; Zamponi et al., 2015), but Ca_V_1.1 subunit was detected in human and rat basal ganglia where it is co-expressed with RyRs in GABAergic neurons (Takahashi et al., 2003).

#### Ca_V_1.2 (CACNA1C)

##### Neurons

Ca_V_1.2 channels account for 89% of all Ca^2+^ currents mediated by L-type VGCCs in the brain (Alves et al., 2019; Enders et al., 2020). In hippocampal neurons, Ca_V_1.2 channels localize to somatodendritic compartment being placed at synapses or extra-synaptically (Joux et al., 2001; Hoogland and Saggau, 2004; Obermair et al., 2004; Tippens et al., 2008; Ortner and Striessnig, 2016), as well as to axons and/or extrasynaptic regions of axonal terminals (Tippens et al., 2008). Within the WM, Ca_V_1.2 channels were identified in the developing rat pioneer axons and the follower axons projecting through the optic nerve, corpus callosum, anterior commissure, lateral olfactory tract, corticofugal fibers, thalamocortical axons, and the spinal cord (Ouardouz et al., 2003; Huang et al., 2012).

Ca_V_1.2 channels open upon membrane depolarization beyond −30 mV, and mediate direct Ca^2+^ entry from the extracellular space into the cytoplasm. In addition, they may act as voltage sensors, transducing membrane depolarization to the RyRs activation and subsequent Ca^2+^ release from the ER *via* the mechanism of Ca^2+−^induced Ca^2+^ release (CICR) (Ouardouz et al., 2003; Micu et al., 2016; Vierra et al., 2019). Clustering and functional coupling of plasmalemmal Ca_V_1.2 channels to RyRs of the ER is mediated by the K_V_2.1 channels (Vierra et al., 2019).

Neuronal Ca_V_1.2 channels are involved in synaptic modulation, propagation of dendritic Ca^2+^ spikes, regulation of glutamate receptor trafficking, CREB phosphorylation, coupling of excitation to nuclear gene transcription, modulation of long-term potentiation, spatial learning, and fear response (Hofmann et al., 2014; Hopp, 2021). During brain development, spontaneous Ca^2+^ transients mediated by Ca_V_1.2 channels regulate neurite growth and axonal pathfinding (Huang et al., 2012; Kamijo et al., 2018). Genetic variations in *CACNA1C* gene are associated with Timothy syndrome, Brugada syndrome, epilepsy, depression, schizophrenia, and autism spectrum disorders (Bhat et al., 2012; Bozarth et al., 2018).

##### Glia

Ca_V_1.2 channels are expressed in cultured astrocytes and mediate Ca^2+^ transients upon direct Ca^2+^ entry and/or subsequent activation of RyRs (D'Ascenzo et al., 2004; Du et al., 2014; Cheli et al., 2016b). Ultrastructural studies found Ca_V_1.2 proteins also in hippocampal astrocytes (Tippens et al., 2008). *In vitro*, Ca_V_1.2 channels contribute to the mechanism of astrogliosis (Du et al., 2014; Cheli et al., 2016b), and in mouse models of Alzheimer's disease, they were detected in reactive astrocyte associated with Aβ-positive plaques (Willis et al., 2010; Daschil et al., 2013).

Ca_V_1.2 mRNA and/or protein are expressed in oligodendrocytes and their progenitors (Agrawal et al., 2000; Paez et al., 2009, 2012; Fulton et al., 2010; Haberlandt et al., 2011; Cheli et al., 2016a; Larson et al., 2016; Marques et al., 2016; Santiago Gonzalez et al., 2017; Paez and Lyons, 2020; Pitman et al., 2020). Ca_V_1.2 channels may regulate proliferation, migration, survival, or differentiation of OPCs, and myelination (Cheli et al., 2015, 2016a; Paez and Lyons, 2020; Pitman et al., 2020). In human cultured OPCs, static magnetic stimulation augmented Ca_V_1.2 mRNA expression, intracellular Ca^2+^ levels, and OPC differentiation (Prasad et al., 2017), suggesting a causal relationship between these processes.

Functional expression of Ca_V_1.2 channels in microglia is still debated (Hopp, 2021). Sequencing data showed no/low CACNA1C expression in microglia (Hammond et al., 2019), and no Ca_V_1.2 was found in cultured microglia even upon stimulation with TNF-α/IFN-γ (Schampel et al., 2017). However, increased immunolabelling for α1C-subunit of L-type VGCCs was observed in reactive microglia during excitotoxicity in rat hippocampus (Espinosa-Parrilla et al., 2015).

##### Expression and Function in MS

Bulk RNA-seq detected increased *CACNA1C* expression in CA lesions (Figure 2, Table 1; Elkjaer et al., 2019; Frisch et al., 2020). The snRNA-seq showed significant *CACNA1C* expression in neuronal and pericyte clusters (Jakel et al., 2019; Tables 1, 2), while low expression in OPCs and astrocyte clusters. In mouse models of MS, application of L-type VGCCs blockers reduces brain and spinal cord WM damage, decreases mitochondrial pathology in nerve fibers, attenuates axonal loss, increases oligodendrocyte survival, and promotes remyelination (Brand-Schieber and Werner, 2004; Schampel et al., 2017; Ingwersen et al., 2018; Zamora et al., 2020). These findings suggest that Ca_V_1.2 channels contribute to damage during MS. However, expression and activity of Ca_V_1.2 channel increased in OPCs within the demyelinated lesions in the mouse corpus callosum after cuprizone treatment (Paez et al., 2012), and deletion of Ca_V_1.2 specifically in OPCs resulted in reduced myelination and lower MBP and MOG expression (Santiago Gonzalez et al., 2017). Hence, activity of L-type channels in oligodendroglial lineage is crucial for remyelination in this MS model, but it is unclear whether oligodendroglial Ca_V_1.2 channels also play a role during MS in humans. Upregulation of Ca_V_1.2 channels in pericytes may reflect altered microcirculation in MS lesions, in analogy to the role of L-type VGCCs in pericytes outside the brain (Hashitani and Mitsui, 2019).

#### Ca_V_1.3 (CACNA1D)

##### Neurons

Ca_V_1.3 channels localize primarily in neuronal cell bodies and dendrites in GM (Hell et al., 1993; Zhang et al., 2005) but were also found in the developing rat optic nerve, corpus callosum (Huang et al., 2012), and axons in spinal dorsal columns of adult rats where they form clusters with RyR2s (Ouardouz et al., 2003). Ca_V_1.3 channels activate at the membrane potential of −55 mV (Lipscombe et al., 2004) and are important players in generating the pacemaking activity and spontaneous firing (Zuccotti et al., 2011). Ca_V_1.3 channels control Ca^2+^-dependent post-burst after-hyperpolarization in CA1 pyramidal neurons, and their activity may trigger Ca^2+^-dependent intracellular signaling pathways (Gamelli et al., 2011; Striessnig et al., 2014). Ca_V_1.3 channels may contribute to the mechanisms of memory because their increased expression correlates with memory loss during aging while their inhibition improves age-related memory deficits (Veng et al., 2003). Deletion of Ca_V_1.3 channels results in increased firing rates of amygdala neurons (probably caused by a reduced slow component of post-burst after-hyperpolarization) and underlies altered fear consolidation in Ca_V_1.3 knockout mice (McKinney et al., 2009). Ca_V_1.3 channels are important for formation of cellular architecture: their various splice variants regulate morphology of dendritic spines while their deletion results in reduced morphology of axonal arbors (Hirtz et al., 2012; Stanika et al., 2016).

##### Glia

Ca_V_1.3 mRNA and/or protein were detected in cultured or freshly isolated rat brain astrocytes; Ca_V_1.3 channels may mediate intracellular Ca^2+^ increase directly and *via* Ca^2+^-mediated activation of RyRs (Latour et al., 2003; Yan et al., 2013; Du et al., 2014; Enders et al., 2020). Ca_V_1.3 expression increases in reactive astrocytes after status epilepticus in mice, suggesting that role in initiation, maintenance, or spread of seizures (Xu J. H. et al., 2007). Yet, other studies have not found Ca_V_1.3 channels in astrocytes (D'Ascenzo et al., 2004).

Ca_V_1.3 channels are expressed in cortical and hippocampal OPCs where they, in concert with other Ca^2+^ channels, may mediate Ca^2+^ entry from the extracellular space and/or trigger CICR from the ER (Haberlandt et al., 2011; Cheli et al., 2015). Knockdown of Ca_V_1.3 reduces Ca^2+^ influx but does not affect expression level of myelin proteins, proliferation, or morphological differentiation of OPCs (Cheli et al., 2015). In the adult rat spinal cord WM, Ca_V_1.3 channels are expressed by APC-positive oligodendrocytes, may mediate oligodendrocyte-axon signaling, and/or contribute to Ca^2+^-dependent injury following trauma (Sukiasyan et al., 2009). Static magnetic stimulation may alter Ca_V_1.3 gene expression level in human cultured OPCs (Prasad et al., 2017), suggesting that external manipulations may be a useful approach to modulate L-type VGCCs in oligodendroglial cells during diseases.

RNA-seq detected *CACNA1D* gene (and its various splice variants) in microglia (Hammond et al., 2019), and its expression increased upon microglia activation (Espinosa-Parrilla et al., 2015). Ca_V_1.3 channels regulate synthesis and release of pro-inflammatory substances from microglia, e.g., NO and TNF-α (Espinosa-Parrilla et al., 2015).

##### Expression and Function in MS

Bulk RNA-seq showed *CACNA1D* upregulation in CA lesions (Elkjaer et al., 2019; Frisch et al., 2020), while snRNA-seq detected significant expression of *CACNA1D* in neuronal clusters (Jakel et al., 2019; Tables 1, 2). Administration of L-type VGCCs blockers resulted in multiple beneficial effects in animal MS models (see above), suggesting that Ca_V_1.3 channels, perhaps in concert with Ca_V_1.2 channels, contribute to tissue damage during MS.

### P/Q-Type VGCCs

#### Ca_V_2.1 (CACNA1A)

The *CACNA1A* gene encodes the pore-forming α1-subunit of P/Q-type (Ca_V_2.1) VGCCs. Sensitivity to ω-Agatoxin distinguishes Ca^2+^ currents mediated by these channels.

##### Neurons

Ca_V_2.1 channels localize on axonal synaptic terminals and play a fundamental role in neurotransmitter release: their direct interaction with the SNARE proteins and synaptotagmin is required for positioning the docked synaptic vesicles near the Ca^2+^ channels for fast vesicular exocytosis (Rettig et al., 1996; Zamponi et al., 2015; Mochida, 2019). Ca_V_2.1 channels are also present at somatodendritic compartments of neurons (Catterall, 2000; Zamponi et al., 2015; Mochida, 2019) where they co-localize with BK and SK channels and provide Ca^2+^ for activation of these channels (Berkefeld et al., 2006; Indriati et al., 2013; Irie and Trussell, 2017). Ca^2+^ enters through the Ca_V_2.1 channels and triggers further Ca^2+^ release from the intracellular stores upon activation of RyRs on the ER (Berkefeld et al., 2006; Indriati et al., 2013; Irie and Trussell, 2017). These mechanisms control neuronal firing even in the millisecond time scale (Irie and Trussell, 2017). Somatodendritic Ca_V_2.1 channels regulate gene expression, local Ca^2+^ signaling, and cell survival (Pietrobon, 2010).

Ca_V_2.1 channels are also present in the WM, i.e., corpus callosum and developing optic nerve (Alix et al., 2008; Nagy et al., 2017). In the optic nerve, Ca_V_2.1 channels are transiently clustered in the axolemma at the sites where the underlying vesicular and tubular elements are fusing with the axonal membrane (Alix et al., 2008). Some of these sites later become nodes of Ranvier, and mutations of the α1A-subunit results in malformation of the nodes of Ranvier (Alix et al., 2008). In the corpus callosum, Ca_V_2.1 channels mediate fast release of glutamatergic vesicles at axon-OPC synapses, and blockade of these channels in slices reduces release at axon-glia synapses by 88% (Nagy et al., 2017).

Ca_V_2.1 channels may play a role in nociception because inflammatory and neuropathic pain is altered in mice with deletion of Ca_V_2.1 channels (Pietrobon, 2010). Mutations in the *CACNA1A* gene underlie familial hemiplegic migraine type 1, spinocerebellar ataxia type 6, and episodic ataxia type 2, and may be associated with increased risk of epilepsy (Pietrobon, 2010; Rajakulendran et al., 2012; Izquierdo-Serra et al., 2020).

##### Glia

RT-PCR detected α1A-subunit in mouse cortical astrocytes in culture, but Ca_V_2.1 channels did not mediate Ca^2+^ entry into astrocytes (Cheli et al., 2016b). Exposure of mouse primary astrocytes to β-Amyloid did not affect Ca_V_2.1 transcript level (Daschil et al., 2014). However, increased expression of Ca_V_2.1 channels was observed in reactive astrocytes after status epilepticus in mice, suggesting their role in initiation, maintenance, or spread of seizures (Xu J. H. et al., 2007). Ca_V_2.1 channels are expressed in hippocampal OPCs, and in pre-myelinating oligodendrocytes of the brainstem (Haberlandt et al., 2011; Barron and Kim, 2019). In brainstem oligodendrocytes, opening of Ca_V_2.1 channels is triggered upon depolarization mediated by glutamate (*via* AMPA receptors) or high K^+^, as well as upon electrical stimulation of axons (Barron and Kim, 2019), suggesting that Ca_V_2.1 channels mediate Ca^2+^ influx into the oligodendroglial cells upon neuronal activity *in vivo*. In this way, neuronal activity may trigger and/or modulate Ca^2+^-dependent signaling in oligodendroglial cells. RNA-seq detected *CACNA1A* gene in microglia (Hammond et al., 2019). Ca_V_2.1 channels may contribute to glioblastoma progression because their inhibition reduced proliferation of glioblastoma cells, although to a lesser extent than blockade of N-type channels (Nicoletti et al., 2017).

##### Expression and Function in MS

Bulk RNA-seq found *CACNA1A* upregulation in CA lesions (Elkjaer et al., 2019; Frisch et al., 2020). The snRNA-seq revealed significant expression of *CACNA1A* transcripts in neuronal and OPCs clusters (Jakel et al., 2019; Tables 1, 2). *CACNA1A* upregulation in MS may reflect the necessity to build new nodes of Ranvier on demyelinated axons within the CA lesions. In oligodendroglial cells, Ca^2+^ entry through Ca_V_2.1 channels may be required for activation of intracellular signaling pathways necessary for differentiation of OPCs and pre-myelinating oligodendrocytes.

#### Ca_V_2.3 (CACNA1E), R-Type VGCCs

##### Neurons

Ca_V_2.3 channels are localized to the dendritic spines and pre-synaptically (Parajuli et al., 2012). Ca_V_2.3-mediated Ca^2+^ currents activate upon strong membrane depolarization and are distinguished by sensitivity to low NiCl_2_ concentrations and SNX-482 toxin. Presynaptic R-type channels play a role in neurotransmitter release (Wu et al., 1999; Gasparini et al., 2001) and synaptic plasticity (Dietrich et al., 2003; Yasuda et al., 2003; Takahashi and Magee, 2009), but their efficiency in triggering neurotransmitter release may be lower compared to P/Q- or N-type VGCCs if they are placed distantly from vesicle release sites (Wu et al., 1999). Dendritic R-type channels are coupled to SK channels and provide Ca^2+^ influx for their activation during excitatory postsynaptic potentials and back-propagating APs (Bloodgood and Sabatini, 2008; Jones and Stuart, 2013). The capacity of dendritic SK channels to promote generation of dendritic Ca^2+^ spikes also depends on Ca_V_2.3 activation (Bock et al., 2019). Besides, Ca^2+^ influx *via* Ca_V_2.3 channels may be necessary for activation of K_v_4.2 channels (Wang et al., 2014). The Ca_V_2.3 channels also form complexes with BK channels, and this functional interaction modulates AP properties and short-term plasticity in hippocampal neurons (Gutzmann et al., 2019). Studies in KO mice revealed that Ca_v_2.3 channels are involved in the mechanisms of sleep modulation, fear response, pain, and seizures (Saegusa et al., 2000; Lee et al., 2002; Weiergraber et al., 2007; Siwek et al., 2014; Zamponi et al., 2015; Wormuth et al., 2016). Deletion of Ca_V_2.3 channels in mice resulted in larger infarct size after middle cerebral artery occlusion *in vivo* and larger Ca^2+^ entry into the cells upon oxygen-glucose deprivation in slices, suggesting that Ca_V_2.3 channels are protective during ischemic tissue damage (Toriyama et al., 2002).

##### Glia

In primary astrocyte cultures, mRNA and Ca_V_2.3 proteins were detected using RT-PCR, Western blotting, immunohistochemistry, and electrophysiological recordings (Latour et al., 2003; D'Ascenzo et al., 2004). During myelinogenesis, oligodendrocytes within WM of the brainstem, cerebellum, and telencephalon transiently express Ca_V_2.3 channels, but their expression strongly decreases into adulthood (Chen et al., 2000). Ultrastructural analysis demonstrated Ca_V_2.3 immunoreactivity in soma and processes of oligodendrocytes, paranodal loops, and loose myelin sheaths (Chen et al., 2000). RNA-seq detected only negligible *CACNA1* expression in microglia (Hammond et al., 2019).

##### Expression and Function in MS

Bulk RNA-seq showed *CACNA1E* upregulation in CA lesions (Elkjaer et al., 2019; Frisch et al., 2020), while snRNA-seq found significant expression of *CACNA1E* transcripts in neuronal clusters (Jakel et al., 2019; Tables 1, 2). The functional role of Ca_V_2.3 channels in MS is unknown.

### T-Type VGCCs

The T-type channels (Ca_v_3) are low-voltage activated Ca^2+^ channels with α1-subunit being encoded by *CACNA1G* (Ca_v_3.1), *CACNA1H* (Ca_v_3.2), or *CACNA1I* (Ca_v_3.3) gene. They are widely distributed in the brain, spinal cord, and DRGs. Ca_v_3 channels activate around V_rest_, show fast inactivation kinetics (Cav3.1 > Cav3.2 > Cav3.3), and mediate tiny Ca^2+^ currents (Perez-Reyes, 2003; Weiss and Zamponi, 2019). Ca_v_3 channels regulate neuronal excitability and play a role during rhythmic AP bursts of thalamic relay neurons, which underlie generation of neuronal oscillations under physiological (sleep) and pathophysiological (epilepsy) conditions (Suzuki and Rogawski, 1989; Astori et al., 2011). Ca_v_3 channels are involved in regulation of nociceptive pathways, sensory processing, hormone, and neurotransmitter release (Weiss and Zamponi, 2019). Mutations in Ca_v_3 genes are linked to neurodevelopmental, neurological, and psychiatric diseases (Lory et al., 2020). Pharmacological non-selective T-type channel blockers are clinically used as antiepileptic drugs and also show anti-nociceptive effects (Zamponi et al., 2015).

#### Cav3.1, Ca3.2, and Ca3.3 (CACNA1G, CACNA1H, and CACNA1I)

##### Neurons

Ca_v_3 isoforms display distinct distribution patterns with prominent somatodendritic expression in thalamic and hippocampal neurons (McKay et al., 2006). Ca^2+^ imaging and pharmacological experiments showed that Ca_v_3.2 and Ca_v_3.3 subtypes located in the AIS influence the generation and the timing of APs (Bender and Trussell, 2009; Kole and Stuart, 2012). In rodent WM, Ca_v_3 transcripts were detected at low level (Aguado et al., 2016), and information on cellular distribution is lacking.

##### Glia

Some studies detected Ca_v_3.1 transcripts and proteins in rat cortical astrocytic cultures (Latour et al., 2003), while others found only scarce Ca_v_3.1 expression in cultured astrocytes (Cheli et al., 2016b; Kim et al., 2018). Divergent findings showed that Ca_v_3.2 immunoreactivity was absent (Chen et al., 2015) or present (Li et al., 2017) in rat spinal cord astrocytes. Ca_v_3.1 and Ca_v_3.2 transcripts were detected in clonal oligodendroglial CG4 cell line (Rui et al., 2020) and in OPCs isolated from mouse cortex (Zhang et al., 2014) or hippocampal slices (Haberlandt et al., 2011). In microglia, RNA-seq did not detect the Ca_v_3 isoforms (Hammond et al., 2019).

##### Expression and Function in MS

Bulk RNA-seq revealed upregulation of Ca_v_3.2 and Ca_v_3.3 genes in CA lesions and upregulation of Ca_v_3.1 in ILs (Elkjaer et al., 2019; Frisch et al., 2020; Table 1). The snRNA-seq detected Ca_v_3.1and Ca_v_3.3 transcripts in neuronal clusters, while it did not detect the Ca_v_3.2 (Jakel et al., 2019; Tables 1, 2). Genome-wide sequencing identified significant association of a Ca_v_3.2 mutation (*CACNA1Hp.R1871Q*) with patients suffering relapsing-remitting MS (Sadovnick et al., 2017). Ca_v_3 upregulation in MS lesions may be triggered by inflammatory mediators and may contribute to axonal dysfunction. Indeed, prostanoids and hydrogen sulfide modulate Ca_v_3.2 expression and function, and increased Ca_v_3.2 channel activity and axonal accumulation is associated with inflammation and pain (Sadovnick et al., 2017; Chen et al., 2018). T-type currents contribute to Ca^2+^-mediated injury of spinal cord WM axons triggered by anoxia (Imaizumi et al., 1999) and to peripheral nerve injury (Watanabe et al., 2015). L/T-type VGCC blocker lomerizine prevents retinal ganglion cell death after diffuse axonal injury (Karim et al., 2006).

Animal studies suggest that Ca_v_3.1 upregulation in IL, a lesion type with complete demyelination and substantial axonal loss, may play a detrimental role. Specifically, the Ca_v_3.1-deficient mice are markedly resistant to EAE induction, and this effect may be mediated by lower production of granulocyte–macrophage colony-stimulating factor (a cytokine implicated in EAE susceptibility) by CNS-infiltrating Th1 and Th17 cells (Wang et al., 2016). The Ca_v_3.1 subunit is a functionally predominant T-type channel in CD4^+^ T cells (Trebak and Kinet, 2019). The Ca_v_3.1-mediated Ca^2+^ increase is critical for calcineurin-NFAT activation driving transcription of cytokines in T cells, and T cells from Ca_v_3.1-deficient mice show decreased IL-17A, IL-17F, and IL-21 production. The development of isoform-specific modulators should help in establishing the differential role of Ca_v_3 subtypes in MS lesions.

### Ryanodine Receptors

RyRs encompass three mammalian isoforms, *RyR1–3*, which form homo-tetrameric channels on the ER. RyRs are highly conductive Ca^2+^ channels: they get activated by Ca^2+^ influx upon plasma membrane depolarization mediating CICR from the ER (Fill and Copello, 2002; Lanner et al., 2010). In the brain, the RyR2s show predominant expression, followed by RyR3s, and then RyR1s (McPherson and Campbell, 1990; Giannini et al., 1995).

#### RyR2 and RyR3

##### Neurons

RyRs localize along ER of neurons, including WM axons (Giannini et al., 1995). They play a role in vesicle fusion, neurotransmitter release, synaptic plasticity, and growth cone dynamics (Giannini et al., 1995; Kushnir et al., 2018). RyRs form complexes with L-type Ca^2+^ channels: RyR1-Ca_v_1.2 and RyR2-Ca_v_1.3 (Ouardouz et al., 2003). WM axons transduce membrane depolarization to Ca^2+^ release from ER, whereby L-type VGCCs gate RyRs, analogous to “excitation–contraction coupling” in muscles (Ouardouz et al., 2003; Stirling and Stys, 2010). Genetic mutations or oxidative stress can render RyRs leaky to Ca^2+^ and promote defective signals as observed in neurodegenerative disorders, heart failure, and muscular dystrophy (Kushnir et al., 2018).

##### Glia

*RYR2* and *RYR3* transcripts, but only RyR3 protein, were found in cultured astrocytes from mouse brain (Matyash et al., 2002; Kesherwani and Agrawal, 2012). *RyR2* transcripts and proteins were upregulated in spinal WM astrocytes after hypoxic injury (Kesherwani and Agrawal, 2012) and SCI (Liao et al., 2016; Pelisch et al., 2017). All *RYR*s subunits were found in rat optic nerve oligodendrocyte cultures (Ruiz et al., 2010), but *RYR3* was selectively expressed in rat cortical OPCs (Haak et al., 2001; Li T. et al., 2018). RyR3s amplify small inward Ca^2+^ currents in astrocytes and OPC, regulating behavior of these cells (Simpson et al., 1998; Matyash et al., 2002; Haberlandt et al., 2011). RyRs mediate stress response in oligodendrocytes, and RyR inhibition attenuated intracellular Ca^2+^ overload following AMPA excitotoxicity (Ruiz et al., 2010). RyR1 and RyR2 mRNAs were detected in adult human microglia, whereas only RyR3 was found in fetal microglia (Klegeris et al., 2007). RNA-seq did not detect *RYR2* and *RYR3* in mouse microglia (Hammond et al., 2019).

##### Expression and Function in MS

Bulk RNA-seq found upregulation of RyR2 transcripts in CA lesions and downregulation of RyR3 in ILs (Table 1; Elkjaer et al., 2019; Frisch et al., 2020). The snRNA-seq revealed significant expression of RyR2 in neuronal clusters and of RyR3 in the astrocyte1 cluster (Table 1; Jakel et al., 2019). RyR subunits probably play a differential role in perturbed intracellular Ca^2+^ homeostasis in WM cells of SPMS brain. RyR2 in CA lesions may contribute to axonal dysfunction because intra-axonal Ca^2+^ overload mediated by RyRs and IP3Rs activates the mitochondrial permeability transition pore and contributes to axonal dieback and degeneration following WM ischemic injury (Ouardouz et al., 2003; Stirling and Stys, 2010; Kesherwani and Agrawal, 2012) and SCI (Stirling et al., 2014; Liao et al., 2016). The RyRs inhibitor ryanodine significantly attenuates mitochondrial dysfunction (Villegas et al., 2014), axonal dieback, and secondary axonal degeneration in injured WM (Thorell et al., 2002; Stirling et al., 2014; Orem et al., 2017). In line, mice with RyR2 gain-of-function mutation exhibit more axonal damage than wild-type controls following SCI (Stirling et al., 2014), while RyR2 knockdown attenuates mitochondrial dysfunction and ER stress and improves functional recovery (Liao et al., 2016).

Functional RyR3s may contribute to astrocyte migration in response to injury, which is important for tissue remodeling and wound healing. In fact, RyR3s control astrocyte motility because astrocytes from RyR3 KO mice display reduced migratory activity (Matyash et al., 2002). Conversely, RyR3 downregulation in ILs may influence the formation of dense astrocytic scar imposing a major barrier to axonal and myelin regeneration. RyR3s also contribute to intracellular Ca^2+^ transients during OPCs differentiation, while RyR3 inhibition prevents OPCs development (Li T. et al., 2018). Interaction between RyRs and NCX in oligodendrocyte processes may represent an amplification mechanism to generate Ca^2+^ transients required for oligodendrocyte differentiation *in vitro* (Casamassa et al., 2016; Hammann et al., 2018; de Rosa et al., 2019; Boscia et al., 2020). However, it remains unclear whether these mechanisms play a role in human MS. The development of selective modulators will help to establish function of RyRs in MS.

### TRP Channels

Transient receptor potential (TRP) channels are tetrameric non-selective cation channels which encompass 30 different types (Nilius and Owsianik, 2011). Upon TRP channel activation, the membrane potential depolarizes, leading to activation or inactivation of voltage-gated ion channels and regulation of Ca^2+^ signaling (Gees et al., 2010). Various intracellular or extracellular stimuli, including chemical and osmotic stress, can trigger activation of TRP channels (Clapham, 2003). TRP channels are involved in pain, regulation of neurotransmitter release, and immune functions. Vanilloid TRP channels (TRPV), melastatin TRP channels (TRPM), and polycystin TRP channels (TRPP) have been detected in WM lesions of patients with progressive MS.

#### TRPV1

##### Neurons

In the CNS, TRPV1 channels are mainly localized on cell bodies and dendritic spines, but also in synaptic vesicles (Goswami et al., 2010). TRPV1 channels are activated by exogenous (i.e., capsaicin) or endogenous (i.e., high temperatures, acid pH, anandamide, 2-arachidonoylglycerol, and lipid metabolites) stimuli (Van Der Stelt and Di Marzo, 2004). They play a role in weight, appetite, and energy homeostasis (Derbenev and Zsombok, 2016; Christie et al., 2018); synaptic plasticity (Gibson et al., 2008; Wang et al., 2020); neuropathic pain (Rivat et al., 2018); and regulation of inflammatory response (Kong et al., 2017).

##### Glia

TRPV1 channels are expressed in astrocytes (Ho et al., 2014), microglia (Sappington and Calkins, 2008), and, to a lesser extent, oligodendrocytes (Gonzalez-Reyes et al., 2013; Marques et al., 2016).

##### Expression and Function in MS

Bulk RNA-seq showed significant TRPV1 downregulation in CA lesions (Figure 2, Table 1; Elkjaer et al., 2019; Frisch et al., 2020), while snRNA-seq barely detected TRPV1 (Table 2; Jäkel and Williams, 2020). The downregulated TRPV1 in CA lesions may influence neural plasticity and glia response both in the hypocellular inactive demyelinated core and in the hypercellular rim filled with activated glia. However, it is unclear whether dysfunctional TRPV1 has pro- and anti-inflammatory roles, and whether it favors or prevents CA lesion expansion and progression, because experimental findings are inconsistent. In rodents, administration of TRPV1 agonists reduced EAE severity (Tsuji et al., 2010), while the TRPV1 antagonist capsazepine, although ineffective for EAE severity (Paltser et al., 2013), reversed the beneficial effects of the endocannabinoid uptake inhibitor (Cabranes et al., 2005). Beneficial effects of TRPV1 may be mediated by its ability to promote micro-vesicle release from microglia, which enhances glutamatergic transmission in neurons (Marrone et al., 2017). However, on the other hand, TRPV1 stimulation induces the pro-inflammatory phenotype of microglia while downregulation promotes the anti-inflammatory phenotype (Hassan et al., 2014; Marrone et al., 2017). TRPV1 also regulates microglia migration, cytokine production, ROS generation, phagocytosis, and death (Kim et al., 2006; Schilling and Eder, 2009; Miyake et al., 2015). Furthermore, TRPV1 mediates migration and chemotaxis of astrocytes, their activation during stress and injury (Ho et al., 2014), and inflammasome activation. The picture becomes even more complex because TRPV1-KO mice show higher lethality during EAE peak but better recovery in the chronic stage (Musumeci et al., 2011). In addition, genetic deletion of TRPV1 in mice resulted in significant protection in the MOG-EAE model, and less severe breakdown of BBB (Paltser et al., 2013). Interestingly, patients with severe MS progression show over-representation of single-nucleotide polymorphisms (SNPs) in the TRPV1 gene (Paltser et al., 2013) that can affect the expression and activity of the channel and cortical excitability, and modulate pain (Xu H. et al., 2007; Mori et al., 2012; Stampanoni Bassi et al., 2019).

#### TRPV6

TRPV6 channels are distinguished by high Ca^2+^ selectivity (van de Graaf et al., 2006) and constitutive activity at low intracellular Ca^2+^ levels and V_rest_ (Vennekens et al., 2000). TRPV6 channels can form homo- or hetero-tetramers. TRPV5–6 are mainly expressed in epithelial and bone cells (Hoenderop et al., 2003).

##### Neurons and Glia

In the mouse brain, TRPV6 channels are expressed in neurons, while transcripts were detected in astrocytes by RNA-seq (Riccio et al., 2002; Nijenhuis et al., 2003; Batiuk et al., 2020).

##### Expression and Function in MS

Bulk RNA-seq found *TRPV6* downregulation in all MS lesion types and in NAWM (Figure 2, Table 1; Elkjaer et al., 2019; Frisch et al., 2020), but snRNA-seq failed to detect *TRPV6* transcripts (Table 2; Jakel et al., 2019). Little is known about the functional role of TRPV6 in brain cells. However, TRPV6 deletion in trophoblasts correlates with altered extracellular matrix (ECM) formation in the labyrinth during pregnancy (Winter et al., 2020). Hence, it will be important to investigate whether TRPV6 downregulation contributes to ECM alterations observed in SPMS lesions and believed to be a key remyelination-inhibiting factor.

#### TRPM2

##### Neurons

TRPM2 channels are found in cell bodies and neurites (Nagamine et al., 1998; Olah et al., 2009) and often co-localize with a marker of dopaminergic neurons (Bai and Lipski, 2010). They are Ca^2+^-permeable sensors of various stimuli (Huang et al., 2020), contribute to synaptic plasticity, and inhibit neurite outgrowth (Sita et al., 2018).

##### Glia

*TRPM2* transcripts are intensely expressed in mouse microglia (Malko et al., 2019), but only at lower levels in astrocytes and oligodendrocytes (Choi et al., 2015; Marques et al., 2016; Falcao et al., 2018; Batiuk et al., 2020; Table 3). TRPM2 plays a critical role in microglia activation and generation of pro-inflammatory mediators, thus contributing to neuropathic pain, brain damage due to chronic hypo-perfusion, neonatal hypoxia–ischemia, and amyloid-beta (Malko et al., 2019).

##### Expression and Function in MS

Bulk RNA-seq showed increased *TRPM2* expression in the ILs (Table 1; Elkjaer et al., 2019; Frisch et al., 2020). SnRNA-seq found *TRPM2* in neuronal, microglia, and ImOLG clusters. The functional role of TRPM2 channels in ILs, lesions that display reduced microglia density, axonal loss, and upregulation of stress response genes (Elkjaer et al., 2019; Frisch et al., 2020), may be related to neuronal and microglia damage. Indeed, TRPM2 channel is upregulated by diverse pathological stimuli (Malko et al., 2019) and is an important element during oxidative stress, mitochondrial dysfunction (Freestone et al., 2009), and neurodegenerative disorders (Chung et al., 2011). Constitutive TRPM2 activation is triggered by ROS and leads to pathological Ca^2+^ signaling and cell death (Eisfeld and Luckhoff, 2007; Naziroglu and Luckhoff, 2008). Knockout of *TRPM2* gene in mice, or blocking the channels with miconazole, improves pathological outcome in EAE and attenuates painful behavior (Melzer et al., 2012; So et al., 2015; Tsutsui et al., 2018). TRPM2-KO mice show reduction of CXCL2 chemokine production by CNS-infiltrating macrophages and suppressed neutrophil infiltration of the brain tissue (Tsutsui et al., 2018). These findings suggest that TRPM2 may represent a promising target in SPMS.

#### TRPP1 and TRPP3 (PKD2 and PKD2L2)

The TRPP(PKD2) channels are encoded by *TRPP1(PKD2), TRPP2(PKD2L1)*, and *TRPP3(PKD2L2)* genes (www.guidetopharmacology.org) and form Ca^2+^-permeable non-selective cation channels. In the mouse brain, *PKD2* and *PKD2L2* transcripts are detected in neurons and glia (Table 3). TRPP1 is present on the ER, primary cilia, and plasma membrane, and TRPP3 is widely expressed in fetal tissues (Guo et al., 2000).

##### Expression and Function in MS

Bulk RNA-seq detected significant downregulation of PKD2 or PKD2L2 in ILs and CA lesions, respectively (Elkjaer et al., 2019; Frisch et al., 2020) (Figure 2, Table 1). SnRNA-seq detected PKD2 transcripts in neuronal and glia clusters but did not detect PKD2L2 (Table 2). It is unclear whether TRPP downregulation in MS lesions is beneficial or detrimental. On one hand, it may be detrimental because TRPP1 and TRPP3 channels are important for maintaining Ca^2+^ homeostasis and contribute to cell proliferation (Xiao and Quarles, 2010; Xiao et al., 2010), while TRPP1 knockdown results in increased susceptibility to stress-induced cell death in kidney epithelial cells (Brill et al., 2020). On the other hand, overexpression of TRPP contributes to apoptosis (Xiao and Quarles, 2010; Xiao et al., 2010), and TRPP1 is upregulated as a direct consequence of ER and oxidative stress during pathological conditions.

### Chloride Channels

ClC channels mediate voltage-dependent transmembrane transport of Cl^−^. They are expressed in plasmalemma and intracellular membranes forming transmembrane dimers (Weinreich and Jentsch, 2001). ClC proteins can function as Cl^−^ channels or as Cl^−^/H^+^ exchangers. ClCs regulate V_rest_ in skeletal muscle, trans-epithelial Cl^−^ reabsorption in kidneys, and intracellular pH and Cl^−^ concentration through coupled Cl^−^/H^+^ exchange in several cell types including brain cells.

#### CIC-2 (CLCN2)

The *CLCN2* gene encodes a voltage- and volume-regulated CIC-2 channel (Chu et al., 1996), essential for efflux of accumulated Cl^−^ and control of cell volume homeostasis. CIC-2 is expressed in neurons and glia (Jentsch et al., 2005) and is upregulated at low osmolarity, cell swelling, and membrane hyperpolarization (Grunder et al., 1992; Clark et al., 1998).

##### Neurons

CIC-2 localizes on inhibitory interneurons and regulates GABA_A_ receptor-mediated synaptic inputs from basket cells (Foldy et al., 2010). Cl^−^ extrusion by ClC-2 following hyperpolarization ensures the maintenance of low intracellular Cl^−^ concentration following synaptic inhibition (Foldy et al., 2010). The link of ClC-2 mutations with generalized epilepsies in humans suggests an important role of ClC-2 in regulating neuronal excitability (Kleefuss-Lie et al., 2009).

##### Glia

Astrocytes express ClC-2 that interacts with AQP4 to regulate Cl^−^ influx and efflux (Benfenati et al., 2007). ClC-2 is expressed in microglia and may regulate cell volume and phagocytosis (Ducharme et al., 2007). In oligodendrocyte lineage cells, ClC-2 positively regulates OPCs differentiation (Jentsch and Pusch, 2018) and transcription factors for myelin genes, thus contributing to myelin formation and WM integrity (Hou et al., 2018).

##### Expression and Function in MS

Bulk RNA-seq showed significant *CLCN2* downregulation in CA lesions (Elkjaer et al., 2019; Frisch et al., 2020; Figure 2, Table 1). SnRNA-seq detected *CLCN2* transcripts in oligodendrocyte clusters, while they were only faintly observed or absent in other clusters (Table 2). Several findings suggest that *CLCN2* downregulation in MS may reflect altered WM integrity and/or contribute to the mechanisms of myelin destruction: first, ClCN2^−/−^ mice exhibit abnormal WM morphology (Blanz et al., 2007); second, loss-of-function *CLCN2* mutations lead to leukodystrophy; third, loss of cell adhesion molecule GlialCAM, which binds to ClC-2 in glia, is associated with leukodystrophy (Jeworutzki et al., 2012; Hoegg-Beiler et al., 2014). Of note, though, is a recent report showing that leukodystrophy fully develops only when ClC-2 is disrupted in both astrocytes and oligodendrocytes (Goppner et al., 2020). It remains to be investigated whether CLC-2 loss in glia contributes to the failure of myelin repair in human CA lesions.

#### CIC-7 (CLCN7)

The *CLCN7* gene encodes for the chloride-proton antiporter ClC-7 localized to lysosomes and crucial for function of osteoclasts and brain cells (Kornak et al., 2001; Jentsch and Pusch, 2018).

##### Neurons and Glia

In mice, neurons and microglia express ClC-7 protein (Kasper et al., 2005; Majumdar et al., 2011; Weinert et al., 2014), while transcripts were found in astrocytes and oligodendrocyte lineage (Falcao et al., 2018; Batiuk et al., 2020). Mutations in the human *CLCN7* gene are associated with osteopetrosis and neurodegeneration (Kornak et al., 2001).

##### Expression and Function in MS

Bulk RNA-seq detected significant *CLCN7* downregulation in CA lesions (Elkjaer et al., 2019; Frisch et al., 2020; Figure 2, Table 1). SnRNA-seq found *CLCN7* transcripts in neuronal and all glia clusters (Table 2). Functional role of ClC-7 under demyelinating conditions is unknown. In neurons, ClC-7 on lysosomes contributes to the function of the endosomal–lysosomal pathway (Poet et al., 2006; Bose et al., 2021). Lysosomal localization of CLC-7 increases during microglia activation, leading to increased lysosomal acidification and Aβ degradation (Majumdar et al., 2011). ClCN7*-*deficient mice display widespread WM atrophy, neuronal loss, microglia activation, astrocytosis, and accumulations of storage material in lysosomes (Kornak et al., 2001; Kasper et al., 2005; Pressey et al., 2010). In SPMS lesions, dysfunctional ClC-7 activity may directly affect the luminal pH and Cl^−^ concentrations and lysosomal protein degradation (Wartosch et al., 2009), which, in turn, may lead to neuronal and glial degeneration in the WM.

### Connexins

Connexins (Cxs) are transmembrane proteins with channel and non-channel functions. Channel functions include the formation of gap junctions (GJs) and hemichannels (HCs) (Saez et al., 2003; Wang et al., 2013; Gajardo-Gomez et al., 2016), while non-channel functions involve adhesion properties and intracellular signaling (Zhou and Jiang, 2014; Leithe et al., 2018). More than 20 Cxs genes have been described in humans, and 11 of them are expressed in the brain (Willecke et al., 2002; Theis et al., 2005). Cxs are essential players in ionic homeostasis, intercellular Ca^2+^ signaling and Ca^2+^ waves propagation, gliotransmission, synaptic transmission and plasticity, brain metabolism, brain–blood barrier development and integrity, and myelination (Takeuchi and Suzumura, 2014). In the WM, GJs are essential for K^+^ buffering in response to neuronal activity, they facilitate transport of nutrients and ions from oligodendrocyte soma to myelin layers and from astrocytes to oligodendrocytes (Bradl and Lassmann, 2010). In the WM, HCs are involved in metabolic coupling and energy supply to neurons, and provide a major pathway for glucose entry into OPCs and oligodendrocytes (Niu et al., 2016).

#### Cx37 (GJA4)

Cx37, encoded by *GJA4* gene, predominantly builds heterotypic GJs with Cx40 and Cx43 in vascular cells and plays an essential role in vasomotor activity, endothelial permeability, and maintenance of body fluid balance (Falcao et al., 2018; Li et al., 2018).

##### Expression and Function in MS

Bulk RNA-seq revealed significant *GJA4* upregulation in ILs (Elkjaer et al., 2019; Frisch et al., 2020), while snRNA-seq showed high *GJA4* expression in pericyte cluster (Jakel et al., 2019; Tables 1, 2). In chronically demyelinated axons, as those within ILs, hypoxia due to imbalance between increased energy demand and reduced ATP production because of mitochondrial dysfunction may drive angiogenesis. However, while providing trophic factors for tissue remodeling, angiogenesis may contribute to hypoperfusion and neurovascular uncoupling (Girolamo et al., 2014). Interestingly, Cx37 knockdown with siRNA in human umbilical vein endothelial cells diminishes capillary branching (Gartner et al., 2012), but Cx37^−/−^ mice develop a more extensive vasculature under ischemic conditions and show enhanced recovery after hind limb ischemia (Fang et al., 2011). In the future, it will be important to investigate whether Cx37 protein contributes to aberrant cerebrovascular and angiogenic responses in human ILs during MS.

### Pannexins

The Pannexin (Px) family consists of three members, encoded by *Panx1, Panx2*, and *Panx3* genes. Pannexins do not form GJ *in vivo* but operate as plasma membrane channels (pannexons) and participate in paracrine and autocrine signaling in brain GM and WM (Sosinsky et al., 2011; Sahu et al., 2014; Dahl, 2015).

#### Px1 (PANX1)

Px1 is permeable to anions, some negatively charged molecules (glutamate, aspartate, and ATP), and fluorescent dyes (Ma et al., 2012; Yeung et al., 2020). Opening of Px1 may be promoted by voltage, increased intracellular Ca^2+^, mechanical stress, extracellular K^+^, oxygen deprivation, caspases cleavage, ATP binding to P2Y or P2X_7_ receptors, activation of α1-adrenergic, NMDA, and thromboxane receptors (Chiu et al., 2018; Dahl, 2018; Whyte-Fagundes and Zoidl, 2018).

##### Neurons and Glia

Px1 is distributed in GM and WM regions, including cerebellum, corpus callosum, and fimbria fornix of mice (Bruzzone et al., 2003) and rats (Vogt et al., 2005). Px1 is expressed in neurons, astrocytes, microglia, oligodendrocytes, vascular cells, and peripheral immune cells (Iglesias et al., 2009; Swayne et al., 2010; Orellana et al., 2013; Good et al., 2018; Lapato and Tiwari-Woodruff, 2018). In neurons, Px1 may be co-expressed with Px2 and is found in cell soma, dendrites, and axons (Cone et al., 2013).

Interaction between Px1 and purinergic signaling deserves special attention because Px1 forms complexes with P2X_7_Rs (Taruno, 2018). Binding of ATP to P2X_7_R triggers opening of Px1 channels with subsequent ATP release (Locovei et al., 2007; Iglesias et al., 2008; Pelegrin et al., 2008; Chiu et al., 2018). ATP signaling involving Px1 channels regulates neurite outgrowth and synaptic plasticity in neurons, while in glia, it underlies intercellular propagation of Ca^2+^ waves, cell differentiation, and migration (Giaume et al., 2021).

##### Expression and Function in MS

Bulk RNA-seq showed significant *PANX1* upregulation in ILs (Table 1; Elkjaer et al., 2019; Frisch et al., 2020), but snRNA-seq did not detect *PANX1* transcripts (Jakel et al., 2019). ILs are lesions with little/no inflammatory activity but with sharply demarcated hypocellular areas of demyelination and axonal degeneration. Px1 activation is known to enable ATP release, and ATP is a “find me” signal promoting chemotaxis of microglia/macrophages to the injury site for fast clearance of dead cells and a molecule important for myelination (Chekeni et al., 2010; Gajardo-Gomez et al., 2016). Hence, *PANX1* upregulation in ILs may be a compensatory mechanism that stimulates glial activity. On the other hand, upregulated Px1 mRNA expression in cerebellum and spinal cord in chronic EAE contributes to WM damage (Lutz et al., 2013). Uncontrolled opening of P2X_7_R-Px1 complex in response to demyelination triggers excessive glutamate and ATP release, altered Ca^2+^ dynamics, excitotoxicity, damage of axons, and myelin (Orellana et al., 2011; Crespo Yanguas et al., 2017). Knockout or blockade of Px1 with probenecid in rodents restrains EAE symptoms and results in reduced inflammation and decreased oligodendrocyte damage (Hainz et al., 2017), suggesting that Px1 activity supports damage during MS. More studies are required to establish how Px1 should be modulated in order to halt neurodegeneration during MS.

### CatSper Channels

#### Catsperg and Caspere

Cation channel of spermatozoa (CatSper) is a highly complex multi-subunit voltage-gated Ca^2+^-permeable ion channel. Four distinct α-subunits (CatSper1–4) and several accessory subunits are encoded by *CATSPER* genes (Qi et al., 2007). The CatSper channel is essential for the activity of sperm flagellum and sperm fertility (Lishko and Mannowetz, 2018). RNA-seq detected only CATSPERG transcripts in mouse neurons, oligodendrocytes, and microglia (Marques et al., 2016; Hammond et al., 2019; Jakel et al., 2019).

##### Expression and Function in MS

Bulk RNA-seq found downregulation of the auxiliary subunit gamma (*CASPERG*) and epsilon (*CATSPERE*) in CA lesions and ILs, respectively (Table 1; Elkjaer et al., 2019; Frisch et al., 2020). SnRNA-seq did not find CATSPERE transcripts and barely detected CATSPERG in neuronal and glia clusters. It is difficult to speculate on the role of CatSper channels in MS lesions because characterization of these subunits is limited to sperm cells, and no data on CatSper protein expression or function in the brain are available.

## Conclusions

Understanding how distinct ion channels regulate CNS ionic homeostasis in WM neurons, axons, glia, and vascular cells under chronic demyelinating conditions is of critical importance for the development of novel therapeutic strategies to prevent neurodegeneration and disability progression and improve functional recovery and repair in MS. Recent Bulk RNA-seq (Elkjaer et al., 2019; Frisch et al., 2020) revealed a considerable number of ion channel genes that are altered in different types of WM lesions of the SPMS brain, particularly in WM CA lesions, a type of lesion that develops in MS patients despite disease-modifying therapy and predicts a more aggressive disease course (Absinta et al., 2019; Elliott et al., 2019). SnRNA-seq found that transcripts for dysregulated ion channels belong to the clusters of neurons, astrocytes, oligodendrocyte lineage, microglia/macrophages, and pericytes (Jakel et al., 2019). The dysregulation of ion channel genes in MS may be detrimental or beneficial for functions of neurons, including interstitial neurons. Intense upregulation of genes encoding voltage-gated Na^+^ channels in CA lesions may reflect the imbalance of Na^+^ homeostasis observed in SPMS brain (Inglese et al., 2010). Conversely, the upregulation of a large number of voltage-gated K^+^ channel genes may be linked to a protective response to limit neuronal excitability. The altered Cl^−^ homeostasis, revealed by the significant downregulation of voltage-gated Cl^−^ channels in MS lesions, may contribute to an altered inhibitory neurotransmission and increased excitability. Depending on the type of alterations, dysregulated ion channels in MS may favor AP propagation and dampen neuronal hyperexcitability or, on the contrary, may contribute to axonal dysfunction and cell death. Altered expression and/or function of ion channels may also influence key properties of glia including proliferation, migration, spatial buffering, cytokine release, cell metabolism, myelin repair, angiogenesis, BBB permeability, and several other important functions.

We described the importance of uniquely dysregulated genes well-known to play a role in WM dysfunction in the MS brain (KCNA1, KCNA2, SCN2A, and SCN8A), or in experimental models of MS (KCNC3, KCNQ3, KCNK2, CACNA1C, CACNA1G, TRPV1, TRPM2, and PANX1). Furthermore, we highlighted the importance of ion channel genes that are uniquely dysregulated in SPMS lesions but have never been previously explored in MS brain. Those genes are expressed in OPC (KCND2, SCN1A, SCN3A, and CACNA1A), ImOLG (KCNQ3), mature oligodendrocyte (KCNH8), microglia (KCNQ3), astrocyte (KCNN3 and RYR3), and pericyte (GJA4 and CACNA1C) clusters of healthy and SPMS brain. It remains to be investigated whether and how the ionic imbalance in different glial cells, particularly oligodendroglia, contributes to impaired recovery and failure of myelin repair.

Several genes, including KCNA1, SCNA8, SCN11A, CACNA1H, PKD2L2 TRPV6, PANX1, and CATSPERE transcripts, were detected in bulk transcriptome (Elkjaer et al., 2019; Frisch et al., 2020), but were not found by snRNA-seq (Jakel et al., 2019). This discrepancy may be explained by several observations: (1) the transcriptional profiling may vary when lesions analyzed by different studies come from different WM regions (Jäkel and Williams, 2020); (2) snRNA-seq analysis lacks information on gene expression in WM axons that may also contain ion channel transcripts; and (3) snRNA-seq only includes RNA transcripts from the nucleus and may therefore lack RNA transcripts from cytoplasm.

Future experiments on dysregulated ion channels predicted by transcriptomic analysis are expected to provide a better understanding of the molecular mechanism of MS progression and may pave the way for the identification of new therapeutic targets to limit lesion expansion, reduce neurological impairment, and stimulate functional recovery.

## Author Contributions

FB, MK, ZI, and ME: writing-original draft preparation. FB and MK: writing-review and editing. FB, MK, and ZI: funding acquisition. All authors have read and agreed to the final version of the manuscript.

## Conflict of Interest

The authors declare that the research was conducted in the absence of any commercial or financial relationships that could be construed as a potential conflict of interest.

## Abbreviations

AIS: axon initial segment
AL: active lesion
AP: action potential
CA: chronic active lesion
Ca^2+^: calcium
Ca_V_: voltage-gated calcium channels
ClC: chloride channels
CNS: central nervous system
COP: committed OPCs
Cx: connexin
EAE: experimental autoimmune encephalomyelitis
EAG: ether-àgo-go
ER: endoplasmic reticulum
GM: gray matter
HC: hemi-channel
IFN-γ: gamma-interferon
IL: inactive lesions
ImOLG: immune oligodendroglia
K2P: two-pore domain K^+^ channels
K_ir_: inward rectifier potassium channel
KO: knockout
K_V_: voltage-gated K^+^ channels
LPS: lipopolysaccharide
MS: multiple sclerosis
Na^+^: sodium
Na_V_: voltage-gated sodium channels
NAWM: normal-appearing white matter
NCX: sodium calcium exchanger
OPCs: oligodendrocyte precursor cells
Px: pannexin
RL: remyelinating lesion
RyR: ryanodine receptor
SCI: spinal cord injury
SPMS: secondary progressive multiple sclerosis
TRP: transient receptor potential
WM: white matter.
